# Recent Advances in Chemistry and Antioxidant/Anticancer Biology of Monoterpene and Meroterpenoid Natural Product

**DOI:** 10.3390/molecules29010279

**Published:** 2024-01-04

**Authors:** Benedict J. Barras, Taotao Ling, Fatima Rivas

**Affiliations:** Department of Chemistry, Louisiana State University, 133 Choppin Hall, Baton Rouge, LA 70803, USA; bbarra3@lsu.edu

**Keywords:** monoterpene, meroterpene natural products, total synthesis, anticancer activity

## Abstract

Monoterpenes and meroterpenes are two large classes of isoprene-based molecules produced by terrestrial plants and unicellular organisms as diverse secondary metabolites. The global rising incidence of cancer has led to a renewed interest in natural products. These monoterpenes and meroterpenes represent a novel source of molecular scaffolds that can serve as medicinal chemistry platforms for the development of potential preclinical leads. Furthermore, some of these natural products are either abundant, or their synthetic strategies are scalable as it will be indicated here, facilitating their derivatization to expand their scope in drug discovery. This review is a collection of representative updates (from 2016–2023) in biologically active monoterpene and meroterpenoid natural products and focuses on the recent findings of the pharmacological potential of these bioactive compounds as well as the newly developed synthetic strategies employed to access them. Particular emphasis will be placed on the anticancer and antioxidant potential of these compounds in order to raise knowledge for further investigations into the development of potential anti-cancer therapeutics. The mounting experimental evidence from various research groups across the globe regarding the use of these natural products at pre-clinical levels, renders them a fast-track research area worth of attention.

## 1. Background

### 1.1. History

Throughout history, herbal medicines have been used to treat various diseases in humans. The earliest documented incidence of patient treatment using these methods dates back to approximately 2600 BC in Ancient Egypt [1]. These medicinal practices utilized a variety of plant extracts to treat various maladies ranging from simple infections to cardiovascular diseases [1]. These naturally derived compounds are still utilized today in a medicinal context as molecular scaffolds or starting points to build new drugs and molecular probes.

In the past 30 years approximately 50% of all approved drugs for cancer treatments were either natural products or derived from a natural product scaffold [2]. Although modern medicine has overcome some of the historical reliance of physicians on natural therapeutic agents, it is estimated that 80% of people worldwide rely on natural-derived medicinal agents as some form of primary care [3]. These compounds still provide an invaluable opportunity to further explore human disease and treatment. Furthermore, as of 2005 less than 10% of the biodiversity on this planet had been biologically evaluated for their therapeutic potential [4] this underscoring the vast potential of these resources. Various recent reviews have highlighted the importance of natural product research in anticancer and antibiotic research for similar natural products not included here [5,6,7,8].

### 1.2. Classification

Terpenes are the largest classification of biologically produced organic molecules accounting for approximately one third of all characterized natural products. These compounds are structurally classified as combinations of monomeric isoprene units because are likely to follow the isoprene rule. In 1953, this rule was proposed to rationalize the biogenesis of terpenoid compounds. The isoprene rule not only clarified the biochemical origin of this class of molecules but also serves as the basis for their classification [9].

**Figure 1:**
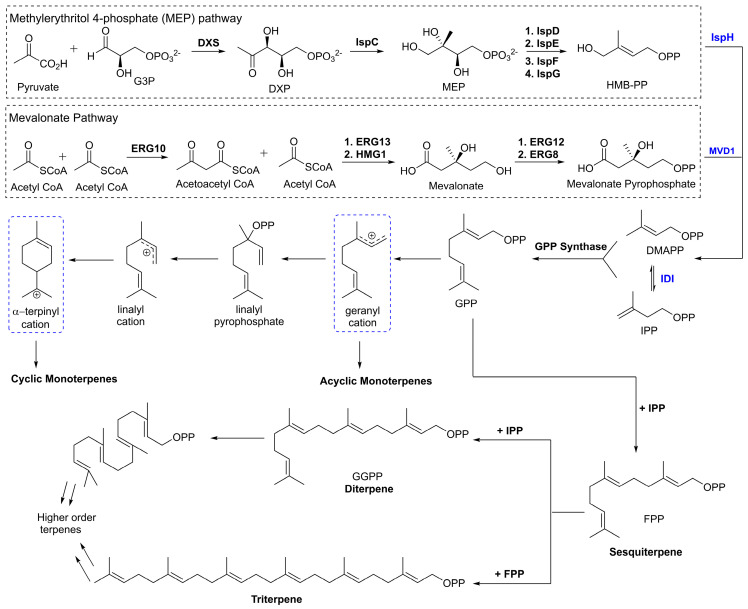
Simplified detailing the convergent biosynthesis of acyclic, monocyclic, and bicyclic monoterpenes highlighting the mevalonate and MEP pathways.

The subgroups of terpenes are defined by the number of isoprene units present in the molecule. Because all terpenes are composed of a number of isoprene units, they will share the hydrocarbon formula (C_5_H_8_)_n_. Activated isoprenoid precursors, isopentyl pyrophosphate (IPP) and dimethylallyl pyrophosphate (DMAPP), polymerize in a variety of combinations through enzymatically catalyzed coordination-insertion, coordinative chain growth and radical ionic mechanisms. These polymerizations afford higher order terpenoid compounds (Figure 1). The various combinations of IPP and DMAPP as well as the potential for rearrangement mechanisms at each step yields a high degree of structural diversity in this class of molecules.

Monoterpenes are the smallest molecules of the isoprenoids with a conserved hydrocarbon formula of C_10_H_16_. There are more than 400 known chemical structures that have been classified as monoterpenes [10]. There have been many monoterpenes isolated and elucidated from natural sources that contain unique and intricate structures each having their own properties in biological systems. These molecules can further be classified as a-, mono-, or bicyclic systems. 

### 1.3. Biosynthesis of Monoterpenes

The methylerythritol 4-phosphate (MEP) pathway is a series of enzymatically mediated steps that was identified in bacteria that yields both of these activated derivatives as shown in Figure 1 [11]. The first committed step of this pathway is the decarboxylation reaction between glyceraldyhyde-3-phosphate (G3P) and pyruvate catalyzed by the enzyme deoxy-d-xylulose-5-phosphate synthase (DXS) [12]. This step results in the production of 1-deoxy-*d*-xylulose 5-phosphate (DXP). Subsequent enzymatic reactions will produce (*E*)-4-Hydroxy-3-methyl-but-2-enyl pyrophosphate (HMBPP) which then undergoes a catalytic dehydration to leave DMAPP and IPP. The mevalonate (MVA) pathway begins with the condensation of three molecules of acetyl CoA to produce mevalonate. Mevalonate is then phosphorylated in two catalytic steps to give mevalonate pyrophosphate. This compound is then decarboxylated to provide IPP which can be isomerized further to produce DMAPP [13].

Following the biosynthesis of the activated isoprene units, a key polymerization reaction takes place between DMAPP and IPP yielding geranyl pyrophosphate (GPP). The enzyme responsible for this reaction, GPP synthase (GPPS), is a highly regulated enzyme that is responsible for the first committed step towards production of all monoterpene compounds [14]. Through subsequent catalyzed reactions two important intermediates emerge as the starting point for further derivatization of monoterpenes. The first, geranyl cation, serves as the substrate for production of most acyclic monoterpenes. From this cationic intermediate a variety of reactions can occur leading to the monoterpenes. The second transition state, α-terpinyl cation, is thought to be the branch point for the synthesis of mono- and bi-cyclic monoterpenes [15].

### 1.4. Meroterpenes

**Figure 2:**
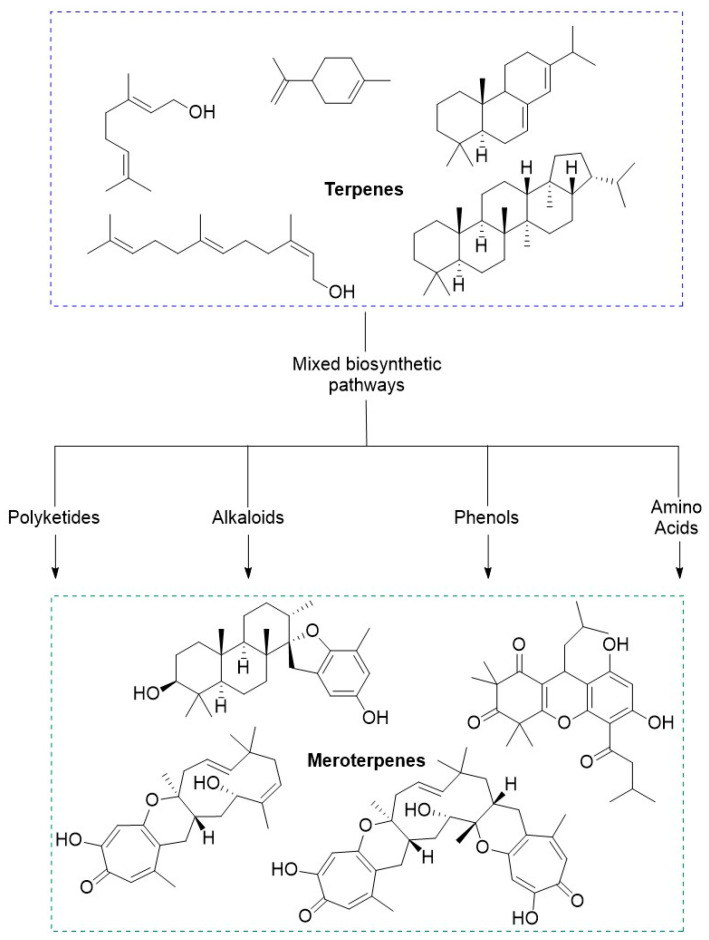
Conceptual depiction of the relationship and biochemical origin of meroterpenoids.

Meroterpenoids are a large group of compounds characterized as being biosynthetically mixed, hybrid molecules. These compounds typically involve the combination of a terpene with polyketides, alkaloids, phenols, or amino acids (Figure 2). The large potential for combination affords this class of molecules with a high degree of structural diversity. Recently these compounds have been gaining attention from chemists and biologists alike for their complex scaffolds and intriguing bioactivities. While many of these compounds have been isolated and identified there is much more to be gleaned from these molecules. Biological evaluations of these compounds have shown their aptitude as potential therapeutic compounds. 

## 2. Biological Activities of Monoterpenes

### 2.1. Acyclic Monoterpenes

Terpenes have been known to be responsible for crucial roles in their parent organism’s biochemical regulation and response to external stimuli. This diverse class of compounds are involved in many biological processes ranging from signaling, hormonal processes, and as biochemical defense mechanisms. In medicine, these compounds have been researched for their biological relevance as potential natural product therapeutics. These natural products have demonstrated antiviral, antifungal, antimicrobial, analgesic, anti-inflammatory, antioxidant, and antitumor properties [16]. This wide array of beneficial biological attributes increases the interest placed on these compounds by medicinal chemists and biologists.

**Figure 3:**
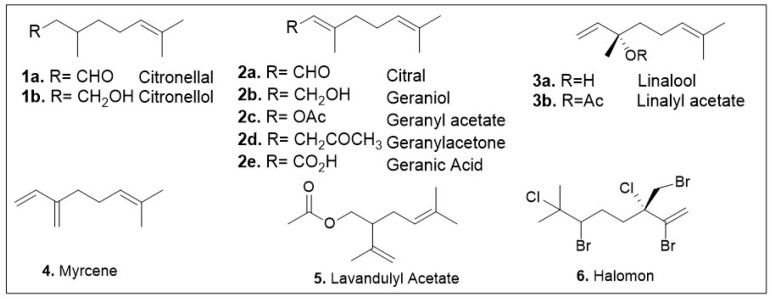
Structures of acyclic monoterpenes that have been identified for their antioxidant or anticancer activities.

The acyclic monoterpene subgroup is quite diverse and only representatives are shown in Figure 3 (**1a**-**6**). Currently, a few of these members have been identified for having antioxidant or anticancer properties, and it remains an active field with a promising future. As it is highlighted here, many of these identified compounds show both antioxidant and anti-cancer activity, but recent representatives are described.

3,7-Dimethyl-2,6-octadienal or citral (**2a**, Figure 3), is an aldehyde-containing monoterpenoid that has been isolated as a volatile compound in essential oils from many different species disbursed across the kingdoms Eukaryota and Plantae [17]. Studies of essential oils containing this natural product have identified citral as having anticancer and antioxidant properties. A recent computational study of the effect of essential oils from Cymbopogon or lemongrass species on the protein network implicated citral as a chemo-preventative compound [18]. Another study utilized proteomic and molecular approaches to show that the essential oil from *Ocimum × africanum* induced apoptosis of human gastric cancer cell line by causing endoplasmic reticulum stress and impairment of the formation of the ribosome. GC-MS analysis of the essential oil showed that citral is a major component comprising 19% of this oils mass [19]. The potential of citral as a medicinal agent was demonstrated using citral-loaded micelles (CLM) as a treatment method for in vitro breast cancer models. This treatment method was tested in multiple breast cancer cell lines, and all showed IC_50_ values of 145, 152, 139, and 130 μM for MCF7, MDA-MB-321, MDA-MB-468, and HCC-1806 respectively [20]. Concerns with these findings are the high concentrations used as well as the lack of data evaluating the citrus compounds cytotoxicity to normal cell lines, therefore further validations studies are warranted.

A recent study conducted in murine models showed this monoterpene’s ability to stave off methotrexate induced lung injury. Treatment of damaged lung tissue with citral resulted in decreased congestion and hemorrhage while no necrotic tissue, alveolar dilation, or interstitial fibrosis was noted in comparison with the methotrexate-treated control [21]. The combined studies suggest citral can mitigate inflammation.

In addition, antioxidant properties of citral were reported in 2021 [22]. The study aimed to determine the mode of action of citral using computational and experimental studies including molecular biology assays such as MTT (3-(4,5-dimethylthiazol-2-yl)-2,5-diphenyltetrazolium bromide) assay, a colorimetric assay for assessing cell metabolic activity, and Chorioallantoic Membrane Assay (CAM) assay, which assess the antiangiogenic properties of the compounds. The studies indicated that citral might affect PPARγ and vascular endothelial growth factor receptors (VEGFR-1 and VEGFR-2). Furthermore, the results of the 2,2-diphenyl-1-picrylhydrazyl (DPPH) radical scavenging assays conducted by this research team confirm this compound’s antioxidant properties [22]. 

Citronellal (**1a**, Figure 3), a prominent constituent of essential oils, has been reported to display anticancer and antioxidant properties [23]. In addition, citronellal was identified as a promising agent against triple negative breast cancer cell lines. Ho showed that a crude extracts of *Citrus hystrix* DC, citronellol, and citronellal significantly reduced cell proliferation and migration by inducing cell cycle arrest in the MDA-MB-231 cell line [23]. The IC_50_ values of the aforementioned extracts were in the millimolar range while those of compounds citronellol and citronellal were 1.16 and 1.41 nM respectively. However, the cytotoxicity of these compounds on normal tissue is of concern as the IC_50_ values of citronellol and citronellal were shown to be 1.96 and 1.81 nM respectively when tested against human monocyte derived macrophages. Independent antiproliferative studies of citronellal using Sulforhodamine B (SRB), MTT, and NRU (neutral red uptake) antiproliferative assays indicate its potential against a triple-negative breast cancer cell model (MDA-MB-231) [24]. The study also conducted in vitro experiments to evaluate citronellal’s activity against LOX-5 (arachidonate 5-lipoxygenase), finding the IC_50_ value to be in the low micromolar range [24]. Furthermore, the compounds efficacy was evaluated using the Ehrlich Ascites Carcinoma (EAC) model where it was found to inhibit tumor cell growth by 46% at 75 mg/kg, well below the lethal dose of 1000 mg/kg [25]. The anti-inflammatory and redox-protective effects of citronellal, indicated by in vitro and in vivo methods were also reported using leukocyte injected mouse models and carrageenan rat models [25]. The redox protective effects were demonstrated by reduced hepatic lipoperoxidation, as well as oxidation of certain protein. Citronellal administration via intraperitoneal route was able to inhibit the carrageenan and arachidonic acid- induced rat hind paw edema models (popular methods for evaluation of anti-inflammatory activity of lipoxygenase inhibitors) [25]. Citronellol (**1b**, Figure 3) has demonstrated notable anticancer properties as well as citronellol-containing essential oils have been reported to display anticancer potential in cell models [21,26]. 

Geranic acid (**2e**, Figure 3) isolated from *Thymus × citriodorus* was recently identified as an anti-inflammatory agent [27]. It is worth noting that in some instances of treatment the required concentrations to elicit a favorable biochemical response proved cytotoxic. While the findings of this study are promising, further work is required prior to the use of the relevant compounds as potential therapeutic agents. Geraniol (**2b**, Figure 3) has been identified as a main bioactive component in essential oils and validated as a pure compound. Geraniol is a major component of Palmarosa, lemon grass, and rose essential oils which have been documented for their beneficial attributes. Geraniol has been shown to protect against cyclophosphamide-induced hepatotoxicity in rat models by modulating expression levels of NFK-β, PPARγ, inducible nitric oxide synthase, and cyclo-oxygenase 2 [28]. Cyclophosphamide is a chemotherapeutic agent with liabilities due to its hepatotoxicity so identification of compounds that can prevent this unfavorable effect are highly desirable. Dosing of rats was done at a concentration of 25 mg/kg once daily one hour after their respective treatments of corn oil (vehicle), or geraniol (100 and 200 mg/kg). The groups treated with geraniol showed lowered expression levels of IL-1β, TNF-α, MDA and other hepatotoxicity markers. The only enzymes shown to be upregulated when treated with geraniol were GSH, and PPARγ. The data shows the promising ability of geraniol to curb some negative side effects caused by the treatment of patients with this effective and well-documented cancer drug. 

Geranyl acetate (**2c**, Figure 3) is a major component of various essential oils and plant extracts that have shown biological activity. A recent study described the cytotoxicity of this compound from the essential oils of kaffir lime callus [29]. This study conducted MTT assays on the breast cancer cell line, T47D and non-cancerous cells Sel Vero. The results of these assays showed a low cytotoxicity in both cancerous and noncancerous cells. The determined IC_50_ values exceeded 100 μg/mL in both cases [29]. These findings suggest that the acetate group makes this compound much more cell permeable.

Geranylacetone (**2d**, Figure 3) has been identified in a number of essential oils and plant extracts that have demonstrated anticancer activities. Recently, geranylacetone was identified as a major component of *Lithocarpus polystachyus* Rehd. a plant with documented anticancer properties [30]. This monoterpene was shown as a major constituent of the volatile compound mixture, present at a concentration of about 283 μg/mL in the distilled sample [30]. An independent study identified geranylacetone as a major component of *Dianella ensifolia* (6% GC-MS) [31]. This study conducted a series of in vitro experiments to understand the antioxidant potential of this oil. A series of biological assays (DPPH, ABTS, and FRAP) were conducted to determine the antioxidant properties of this essential oil (EO) in comparison with positive control BHT (butylated hydroxytoluene). Geranylacetone is also able to react as a free radical scavenger. Both geraniol and geranylacetone have been demonstrated to serve as free radical scavengers, albeit at high concentrations of 4–8.0 mM. DPPH radical scavenging assay showed that geranylacetone after 15 min indicated a modest dose-dependent effect [32].

Halomon (**6**, Figure 3), a halogenated acyclic monoterpenoid, has been reported to be active against the non-small cell lung carcinoma cell model (NSCLCN6-L16) and colon cancer cell model (HCT-116) in vitro. Furthermore, in vivo studies have demonstrated the anticancer activity of this compound against U251 brain cancer. Halomon showed cytotoxicity against many cancer subtypes, including renal-, brain-, and colon-derived solid tumor cell lines. It also showed that this monoterpenoid acts as an inhibitor for DNA methyltransferase-1 with an IC_50_ value of 1.25 μM [33], which decreases the expression of tumor suppressor genes. Preliminary in vivo studies evaluated the biological effect of Halomon via intraperitoneal injections on U251 brain cancer xenograft model. This model involves the implantation of cancer cells into immunocompromised rats; the tumor is allowed to grow, and then the effects of a treatment are evaluated in vivo. It was demonstrated that five daily doses of 50 mg/kg had a 40% success rate in treatment [33].

Anticancer and antioxidant activity have also been demonstrated with lavandulyl acetate (**5**, Figure 3) which is found in essential oils and plant extracts such as *Lavandula angustifolia* (lavender oil). GC-MS indicated that lavandulyl acetate is found in 23 percent (*w*/*w*) of the overall mixture on average of this essential oil [34]. The essential oil was evaluated on several assays including cytokine cellular response, LPS-stimulated HaCaT Keratinocytes, and LPS-prestimulated human monocyte derived macrophages (hMDMs). A dose dependency was observed between lavandulyl acetate in the oil and a potent anti-inflammatory and pro-regenerative response [34]. The studies indicate that this compound might hit several targets as these biological processes involve different pathways. The pharmacological effects of lavender oil depend on the concentration of lavandulyl acetate as independent studies have also shown other biological activities for this essential oil [31]. 

Linalool (**3a**, Figure 3), a chiral acyclic monoterpenoid, has demonstrated anticancer and antioxidative properties [35,36]. The anticancer activity of the essential oil from *Ocimum forskolei* Benth with 28–10% of linalool has been reported [37]. The oil’s IC_50_ values were shown to be close to 17, 7, 5, and 25 μg/mL for MCF7, HT29, HCT116, and MRC5 respectively. A recent study done found that linalool displays antiproliferation effects on breast cancer cell lines MCF-7 and MDA-MB-231. This research demonstrated that at 100 μM concentration of linalool there was approximately 55 and 65 % cell viability for MCF7 and MDA-MB-231 respectively after 24 h [35]. The determined IC_50_ values of linalool were a mild 588 and 480 μM for MDA-MB-231 and MCF7 respectively [35]. Unfortunately, no data was provided for normal cells, which calls for further studies to experimentally determine therapeutic index and its potential as anticancer agent. 

Linalool has demonstrated antioxidative properties in a study from *Plumeria alba* [38]. The essential oils from this plant have been shown to have antioxidant properties. Gas Chromatography-Mass Spectroscopy studies identified that linalool composes about 24% of the active essential oil. The samples prepared from the plants flowers gave IC_50_ values of 370, 1014, and 1943 μg/mL for the H_2_O_2_ scavenging assay, DPPH radical scavenging assay, and thiobarbituric acid reactive substances (TBARS) assay respectively [38]. In addition, in vivo studies in hemiparkinsonian rat models indicated neuroprotective effects. Linalool treatment demonstrated neuroprotectivity in the form of anti-inflammatory and antioxidative action in dosages as low as 50 mg/kg [39]. Through the observed behavioral and neurochemical effects linalool indicated neuroprotective activity presumably through its antioxidative properties [39]. Linalyl acetate (**3b**, Figure 3) has been well documented to have favorable bioactivities. Anticancer properties have been associated with essential oils from *Lavandula angustifolia, Lavandula officinalis, Lavandula latifolia*, and bergamot [40,41,42,43,44], all of which contain linalyl acetate in concentrations as high as 44%. A study conducted on the lavender essential oils from *Lavandula angustifolia* demonstrated an IC_50_ values of 0.36% (*v*/*v*) against HaCaT cell line in an MTT assay [43]. Additionally, there have been reports of linalyl acetate associated antioxidant activity in essential oils of *Salvia sclarea*, and *Myrtus nivellei*, across different regions [45,46]. The essential oil attained from *Myrtus nivellei* demonstrated an IC_50_ value of 2.25 mg/mL [46].

Myrcene (**4**, Figure 3) has been shown to display anticancer bioactivities when used as a mixture in the essential oils from *Mentha spicata*, *Matricaria chamomilla*, and *Citrus pseudolimon* [47,48]. The IC_50_ values of the essential oil from *Matricaria chamomilla* were demonstrated to be between 200 and 320 μg/mL for cancer cell lines A549, MCF7, and PC3. Meanwhile the IC_50_ values for the *Mentha spicata* essential oils were 672, 708, and 206 μg/mL for cell lines A549, MCF7, and PC3 respectively [47]. 

It has been documented that plant essential oils with high concentrations of myrcene have antioxidant potential in biological systems. Essential oils from *Hedyosmum purpurascens, Pinus halepensis*, *Salvia officinalis, Cymbopogon citratus, Piper eriopodon*, and *Prangos trifida*, all having high concentrations of myrcene, have demonstrated antioxidant activities [49,50,51,52,53]. Myrcene has been demonstrated to inhibit oxidative stress by down regulating pro-inflammatory cytokines and inflammatory mediators [54]. This compound’s ability to decrease the oxidative stress induced by rotenone was demonstrated by the significant reduction of pro-inflammatory cytokines upon myrcene-rotenone co-treatment. While monitoring expression levels of inflammatory markers a dramatic decrease in expression was noted in iNOS and COX-2 [54].

### 2.2. Cyclic Monoterpenes

**Figure 4:**
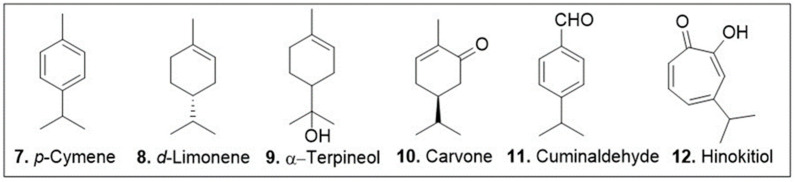
Selected cyclic monoterpenes with reported antioxidant or anticancer activities.

The cyclic monoterpenes **7–12** (Figure 4) have a strong background in biological activities as they are common members of essential oils used in ethnopharmacology [55]. The natural product *p*-cymene (**97**, Figure 4) is an aromatic, cyclic monoterpene that has been demonstrated to have antioxidant properties in vivo. A study conducted in diabetes induced Wistar rats demonstrated this compound’s ability to upregulate mTOR, AKT, and phospho-AKT enzymes all of which being crucial regulators of oxidative stress [56]. The diabetes induced rats were dosed with either 55 mg/kg of metformin, an approved type II diabetes medication, or 25, 50, 100 mg/kg of *p*-cymene. All groups were successful in increasing the expression of these key oxidative stress regulatory enzymes [56]. It is worth noting that although the 25, and 50 mg/kg groups were less effective than metformin, the 100 mg/kg group was able to upregulate these enzymes to a comparable degree as the metformin group [56]. 

*d*-Limonene (**8**, Figure 4) is a chiral, cyclic monoterpene that has been shown to have both anticancer and antioxidant properties. A study conducted in vivo animal models showed this compound’s ameliorative effect on chloroform induced cardiotoxicity by lowering oxidative stress, inflammatory and cardiac markers [57]. The study monitored the change in cardiac injury markers whose expression was increased by CCl_4_; following two weekly doses of 200 mg/kg of *d*-limonene approximately 50% reduction in serum levels of cardiac inflammatory markers IL-6, Hs-CRP, and TNFα was noted [57]. An independent study observed the effects of *d*-limonene in cancer therapy. This work used *d*-limonene for its antioxidant effects in a co-treatment study with doxorubicin [58]. The designed nanoparticle delivery system showed a strong decrease in cell viability against HepG2 human liver cancer cell line and a decrease in cytotoxicity of the LX2 normal cells [58]. Unfortunately, there was no further biological evaluation of this treatment system to further support the hypothesis of the mechanism of action with the doxorubicin-*d*-limonene nanoparticle delivery systems. 

Carvone (**10**, Figure 4) is a chiral, cyclic monoterpenoid present in large quantities in caraway and spearmint essential oils that has been isolated identified for its anticancer and anti-oxidative properties. A study on the anticancer effects of carvone on myeloma cells (KMS-5) indicated that this meroterpenoid induces apoptosis through inhibition of the p38 MAPK signal transduction pathway [59]. It was found that the IC_50_ of carvone against this cell line was 20 μM [59]. Apoptosis was identified as the mode of action for this treatment by DAPI, AO/EB, and V/PI staining assays carried out at concentration of 20 μM carvone. Furthermore, western blot analysis demonstrated the decrease in expression of p-P38, a dual regulator of apoptosis, in response to carvone treatment at 20 μM leading to the conclusion that this compound may inhibit the expression of this regulatory enzyme [59]. An independent study investigated this compound’s effects in alleviation of doxorubicin induced cardiotoxicity. The IC_50_ value of carvone on the MCF7 cell line was determined to be 14 μM while in the healthy cell line H9C2 this monoterpenoid was nontoxic up to 200 μM [60]. In vivo analysis of male BALB/c mice was done to evaluate the synergistic effects of carvone during concomitant treatment with doxorubicin. These experiments showed that the myocardial disorganization and degeneration imposed by 20 mg/kg intraperitoneal (i.p) injection of doxorubicin was alleviated when i.p. injection of 75 mg/kg carvone was done one hour prior to doxorubicin treatment [60]. Identical injection of 150 mg/kg carvone resulted in no notable tissue generation upon histological analysis [60].

Cuminaldehyde (**11**, Figure 4) is an aromatic, cyclic monoterpenoid with anticancer biological properties. Research done on cuminaldehydes effects on human colorectal adenocarcinoma (COLO 205) cells showed that this compound induces cell death through topoisomerase I and II inhibition [61]. An in vitro evaluation of cuminaldehyde treatment of COLO 205 cells demonstrated a notable decrease in cell growth and increase in cytotoxicity at μM concentrations. After 24 h of cell treatment with 40 μM cuminaldehyde there was a 50% decrease in cell viability. This compound was further tested against topoisomerase I and II to show the inhibitory relationship between these molecules. The degradation of monomeric DNA in the presence of 5–20 μM cuminaldehyde indicates the interaction between this monoterpenoid and the nuclear proteins. Furthermore, tumor reduction capacity was demonstrated using a COLO 205 xenograft in a nude mice model. Daily subcutaneous injection of 10 and 20 mg/kg cuminaldehyde resulted in 48 and 69 % tumor reduction respectively [61] with no notable loss in body weight. Although these findings are promising the lack of comparative data with healthy cell lines makes it difficult to evaluate this compound for its efficacy as a potential therapeutic. 

Hinokitiol (**12**, Figure 4) is a tropolone related, cyclic monoterpenoid that has been reported to show activity in anti-inflammatory and antioxidation processes while simultaneously promoting apoptosis and autophagy in cancer cells [62,63]. Western blot analysis of B16F10 murine melanoma cell line and 4T1 murine mammary carcinoma cell line showed this natural products potential of decreasing expression of heparanase, an enzyme known for its pro-metastasis effects in concentrations of 1 to 1250 nM. Furthermore, this research conducted in vivo studies on C57BL/6J and BALB/c mice. The results showed an inhibition of tumor metastasis and prolonged survival [62]. While these results are promising, the study did not analyze the effects of the monoterpene as a form of administered medication and instead injected B16F10 and 4T1 cells that had been pretreated in 1250 nM concentrations of hinokitiol into the mice. An independent study analyzed the cellular response of human osteosarcoma cell lines to treatment of hinokitiol in vitro.

Cell viability assays of U2OS and MG-63 human osteosarcoma cell lines showed a concentration dependent decrease in cell viability with hinokitiol treatments the 10–80 μM concentration range [63]. Furthermore, this research conducted western blot analysis of MG-63 cells showing that with treatment of hinokitiol results in PARP and caspase-3 cleavage both of which being known mediators of apoptosis. Cleavage of PARP was noted after treatment of hinokitiol in concentrations as low as 20 μM and demonstrated a concentration dependent increase through 80 μM [63] suggesting this compound induces programed cell death. Unfortunately, the study did not analyze any of the aforementioned phenomena on healthy cell lines.

## 3. Chemistry and Biological Activities of Selected Meroterpenoids

**Figure 5:**
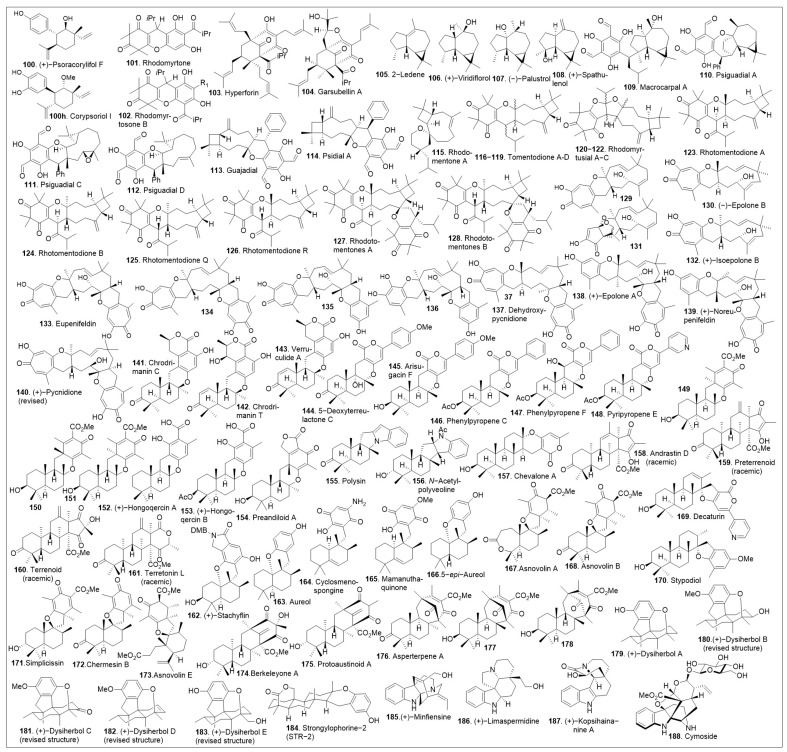
Structures of representative meroterpenoids.

Meroterpenoids are a large group of compounds characterized as being biosynthetically hybrid molecules, which typically involve the combination of a terpene with polyketides, alkaloids, phenols, or amino acids. The large potential for variations in the combination of synthons provides a class of molecules with a high degree of structural diversity. Recently, these natural products have gained attention from chemists and biologists alike as there are new chemical applications and synthetic strategies to access these complex molecular scaffolds. While many of these compounds have been isolated and identified there is a new need for larger quantities to pursue biological studies. Biological evaluations of these compounds have shown potential for therapeutic development, many of these intriguing meroterpene structures have already been reviewed [64] and they are not included in this review. Specific synthetic lessons and innovative approaches are highlighted for the members of this meroterpene family, particularly natural products **100**–**188** (Figure 5). This diagram highlights the complexity and diversity of these natural products containing 3 to 6 rings, which also includes an increase in ring size within this family of natural products. 

**Scheme 1:**
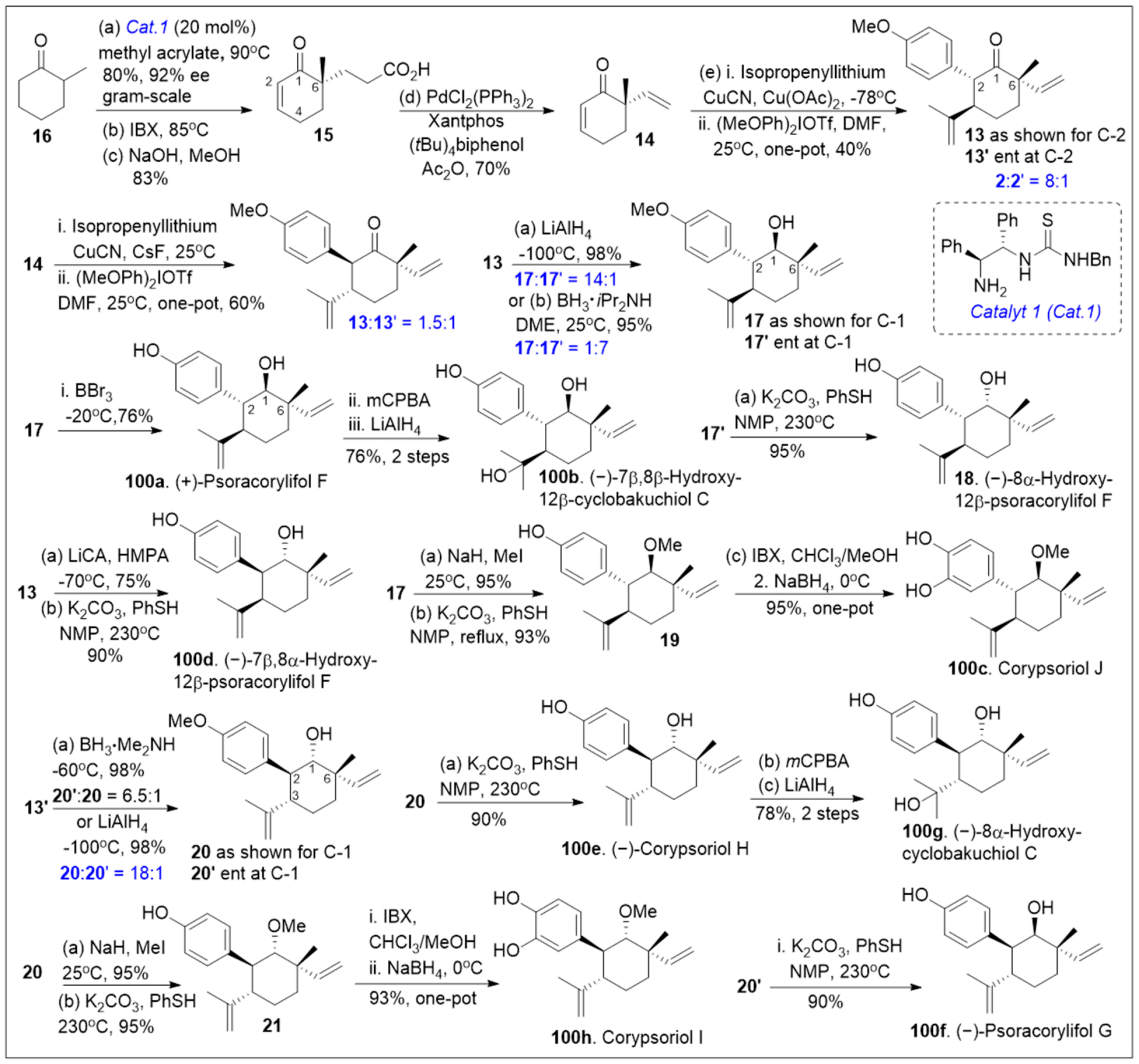
Total synthesis of several meroterpenoids from *Psoralea corylifolia*.

Total syntheses of small molecules can be challenging as insufficient functional groups are available to facilitate carbon-carbon bond formation and/or introduce chirality. Enantioselective divergent total syntheses for several meroterpenoids from *Psoralea corylifolia* have been recently reported [65]. The synthesis commenced with the readily available 2-methylcyclohexanone **16** in gram-scale, which was coupled with methyl acrylate mediated by thiourea catalyst (Cat. 1, Scheme 1) followed by treatment with 2-iodoxybenzoic acid (IBX) to oxidatively generate the corresponding α,β- system and subsequent saponification furnished acid **15**. The newly formed compound **15** was subjected to catalytic PdCl_2_(PPh_3_)_2_ mediated intramolecular decarboxylative vinylation to establish the desired carbon quaternary center of enone **14**. Compound **14** was then subjected to a 1,4-Michael addition reaction in the presence of copper and quenched with an aryl electrophile. This installs the α-arylation reaction sequence yielding diastereomers **13** and **13’**, with compound **13** being predominant in 8:1 d.r. and can be separated by column chromatography. To improve the yield and favor a closer 1:1 ratio, Cesium fluoride was used instead of the copper acetate ligand which provided a 20% increase in yield. When applied properly, strategic conjugate addition reactions, when applied properly can expedite syntheses by forming carbon-carbon bonds efficiently, leading to improved overall yields. Compound **13** was subjected to reduction of the C-1 ketone with LiAlH_4_ or amine borane reagent to enrich one diastereomer over the other one providing alcohols **17** and **17’** in either 14:1 or 1:7 respectively. Compound **17** can be deprotected by BBr_3_ to yield the natural product (+)-psoracorylifol F (**100a**) in good yield, which was subjected to regioselective *m*CPBA epoxidation, and subsequent LiAlH_4_ reductive opening of the epoxide provided (−)-7β,8β-hydroxy-12β-cyclobakuchiol C (**100b**). Aromatic methoxy deprotection of compound **17’** by thermal treatment in the presence of the thio-anion led to the natural product (−)-8α-hydroxy-12β-psoracorylifol F (**18**). Compound **13** was exposed to a three-step synthetic sequence strategy, α-epimerization, carbonyl reduction via NaBH_4_ treatment, and heat-mediated thio-anion methyl deprotection of the phenol provided natural product (−)-7β,8α-hydroxy-12β-psoracorylifol F (**100d**). Alternatively, compound **17** was treated with MeI to protect the secondary hydroxyl group, and the aromatic methoxy group was revealed under thio-anion reaction conditions to yield compound **19** in excellent yields. The resultant compound **19** was oxidized at the alpha phenol with IBX followed by reduction with NaBH_4_ of the newly formed ortho-diketone to provide the natural product corypsoriol J (**100c**). In parallel efforts, compound **13’** was subjected to carbonyl-reduction followed by LiAlH_4_ or amine borane reagent to enhance the diastereoselectivity yielding alcohols **20** and **20’** in 6.5:1 or 18:1 ratio respectively. Deprotection of compound **14** mediated by thio-anion treatment under heat led to natural product (−)-corypsoriol H (**100e**), which upon two-step sequence epoxidation mediated by *m*CPBA and subsequent reductive opening of the epoxide with LiAlH_4_ provided (−)-8α-hydroxy-cyclobakuchiol C (**100g**). In a similar fashion, intermediate **20** was subjected to methyl-protecting group maneuver via intermediate **21**, followed by one-pot oxidation mediated by IBX and subsequent reduction of the newly formed ortho-diketone. This, mediated by NaBH_4_ yielded corypsoriol I (**100h**). Finally, methyl deprotection of compound **20’** mediated by thermal treatment in the presence of thio-anion yielded (−)-psoracorylifol G (**100f**). The short and efficient synthetic strategy enables access to a large number of natural products, which can be further investigated for their biological properties. 

**Scheme 2:**
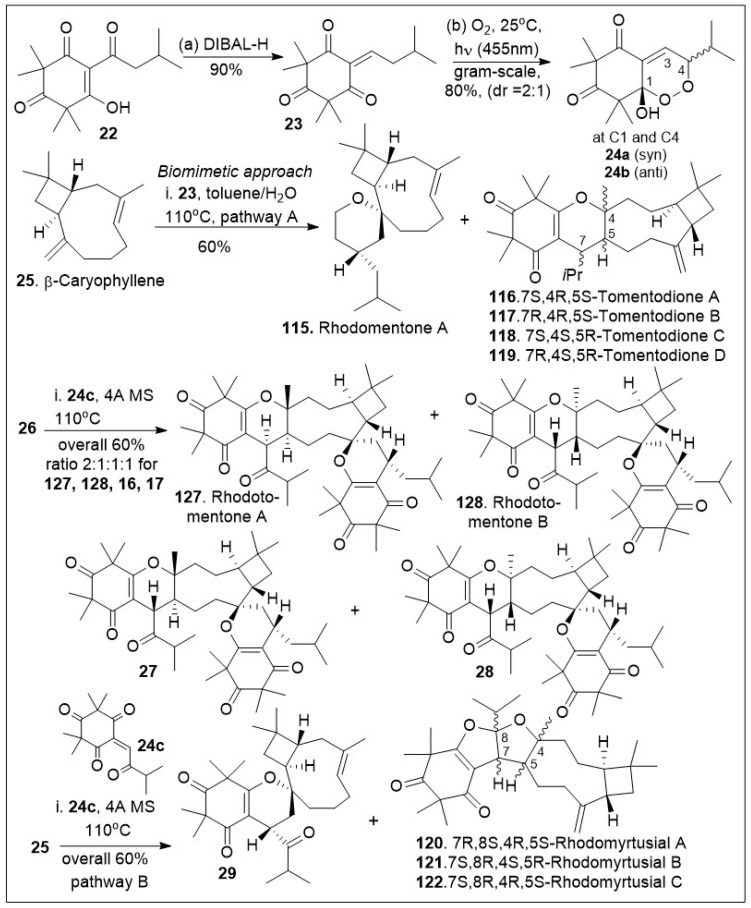
Biomimetic-inspired synthesis of caryophyllene-derived meroterpenoids (part 1).

Rhodomyrtone belongs to a large and diverse family of tricyclic meroterpenoid systems isolated from *Rhodomyrtus tomentosa* [66,67,68,69,70]. This set of compounds has been shown to have strong antibacterial activity, acting primarily against gram-positive strains. In addition, the combined extracts of *Rhodomyrtus tomentosa* have shown anti-cancer and anti-tumoral effects in various models [66,67]. The synthesis of the unusual caryophyllene-derived meroterpenoids (CDMTs) including rhodotomentone A (**127**) and rhodotomentone B (**128**) was reported via a biomimetic pathway [68,69,70], (Scheme 2). The synthesis began from the natural product leptospermone (**22**), a commonly occurring acylphloroglucinol, which was subjected to dehydration mediated by DIBAL-H, yielding the dearomatized *ortho*-quinone methide (*o*-QM) intermediate **23** in gram-scale. This maneuver was followed by a [4+2] Diels-Alder photo-cycloaddition reaction with singlet oxygen, generated under blue LED (455 nm) radiation to provide endoperoxide intermediates **24a** and **24b** in 1:1 ratio. The formed *o*-QM intermediate **23** was subsequently subjected to hetero-Diels-Alder (HDA) reaction with β-caryophyllene **25** to produce a mixture of rhodomentone A (**115**), and the related natural products, tomentodione A, B, C and D (**116**, **117**, **118**, and **119**). Rhodomentone A (**115**) can be treated with endoperoxide **24b** to undergo the key Kornblum-DeLamare rearrangement and Diels-Alder cycloaddition reaction sequence under thermal reaction conditions. This produces rhodotomentone A(**127**) and B(**128**), along with two other rhodotomentone diastereoisomers, compounds **27** and **28**. Additionally, endoperoxide **24b** was reacted with β-caryophyllene (**25**) to undergo the Kornblum-DeLamare rearrangement and Diels-Alder cycloaddition reaction sequence under thermal conditions to generate rhotomentodine A, B, Q and R (**123**, **124**, **125** and **126**), along with rhodomyrtusial A, B and C (**120**, **121** and **122**) and compound **29 [70]**. 

**Scheme 3:**
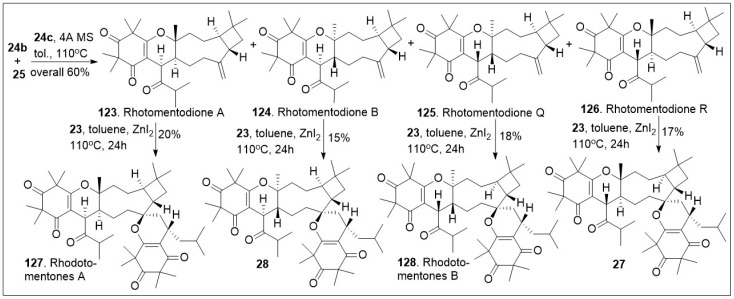
Biomimetic-inspired synthesis of caryophyllene-derived meroterpenoids (part 2).

In an efficient approach (Scheme 3), CDMTs **123**, **124**, **125** and **126** were easily converted to the corresponding natural products rhodomentone A (**127**), **28**, rhodomentone B (**128**) and **27** respectively. This was carried out under hetero-Diels-Alder (HDA) reaction conditions, requiring Lewis acid, ZnI_2_ and heat, in relatively modest yields. The synthetic approach offers rapid access to impressively complex structures [70].

**Scheme 4:**
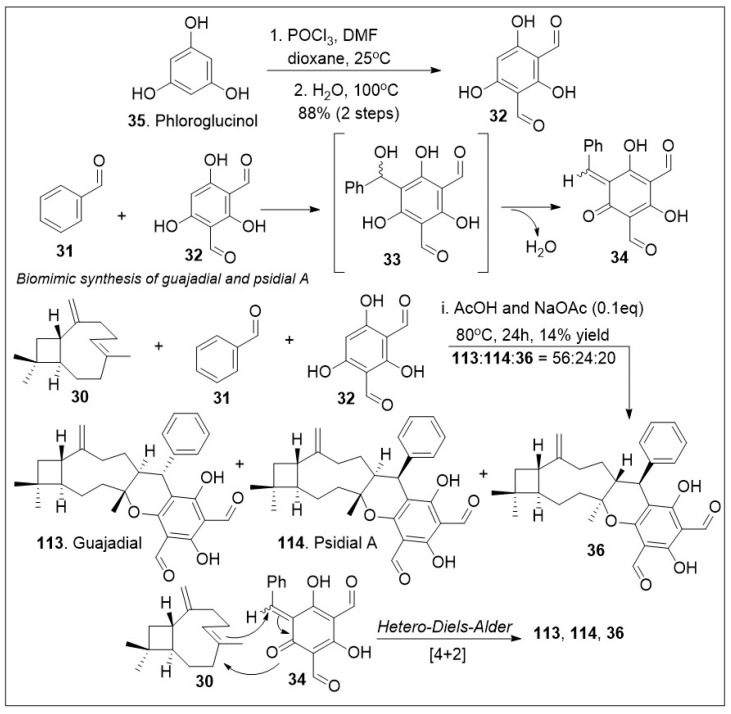
Biomimetic synthesis of guajadial and psidial A.

The meroterpenoids macrocarpals and psigudials isolated from *Psidium guajava* a food crop widely abundant in tropical regions has been reported to display an array of medicinal properties [71,72]. These compounds have displayed significant biological properties as anti-HIV and anti-bacterial agents. To further evaluate their properties, access to sufficient quantities of these compounds is required. The application of a hetero-Diels-Alder (HDA) reaction in meroterpenoid synthesis is frequently utilized due to the likely probability that a similar pathway is taking place in nature to generate these natural products [72]. A short biomimetic-inspired synthesis to guajadial **113** and compound **30** was recently reported (Scheme 4). The synthesis commenced with a Knoevenagel condensation between benzaldehyde **31** and diformylphloroglucinol **32** providing compound **33** for in situ generated *o*-quinone methide **34**. The HDA cycloaddition reaction between caryophyllene **30** and *o*-QM **34** led to guajadial **113**, psidial A **114** and cycloadduct **36** [72].

**Scheme 5:**
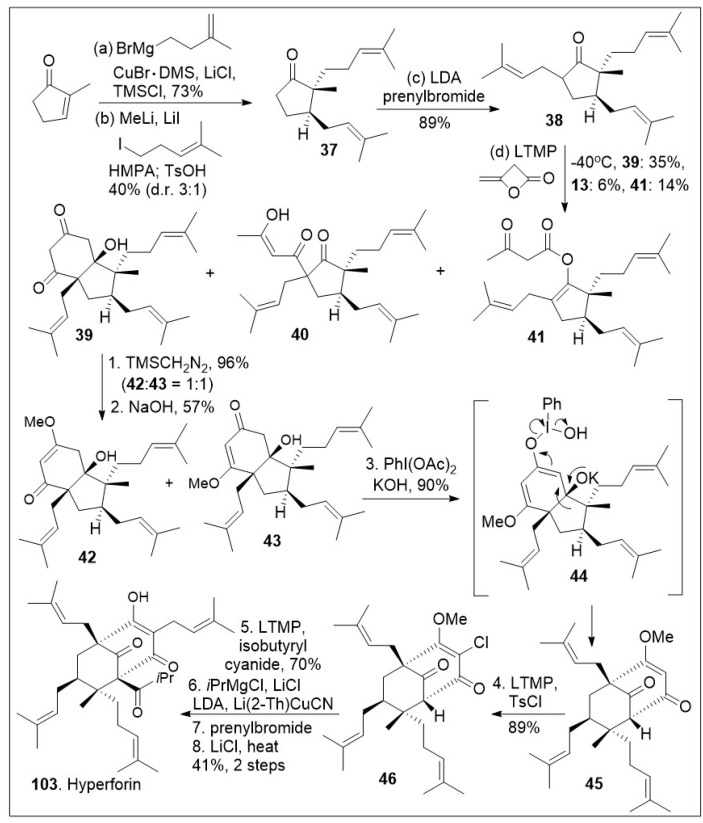
Total synthesis of hyperforin.

Hyperforin-like structures are a large family of meroterpenes that share a challenging molecular caged motif. A recent innovative synthetic strategy based on an annulative approach of polycyclic polyprenylated acylphloroglucinol (PPAP) meroterpenoids with 3,5-dimethylorsellinic (DMOA) was reported as shown in Scheme 5 [73]. The synthesis commenced with the readily available methylcyclopenenone synthon, which was subjected to sequential 1,4-copper-mediated conjugate addition reaction, namely formation of the lithium enolate followed by alkylative enolate trapping to generate the substituted cyclopetanone **37**, which was subjected to further α-alkylated to furnish the highly substituted cyclopetanone **38**. Next, cyclopetanone **38** was subjected to the key annulation reaction with β-lactone diketene, leading to diketone **39** as the major product, along with C-acylated product **40** and O-acylated product **41**. Diketone **39** was further methylated with trimethylsilyldiazomethane to generate the vinylogous ester isomers **42** and **43**. Compound **43** was subjected to oxidative rearrangement mediated by hypervalent iodine to provide product **45** with bicyclo[3.3.1]nonane ring system via intermediate **44**. Compound **45** was subjected to a subsequent chlorination reaction to provide halogenated compound **46**. Finally, a deprotonation/prenylation alkylation, acylation reaction sequence was used to introduce the last isoprenyl group generating the most challenging quaternary center. This was followed by deprotection of the methoxy group to yield hyperforin (**103**) in modest yields. The short strategy is flexible enough to generate a potential diverse library of derivatives for future biological evaluations. 

**Scheme 6:**
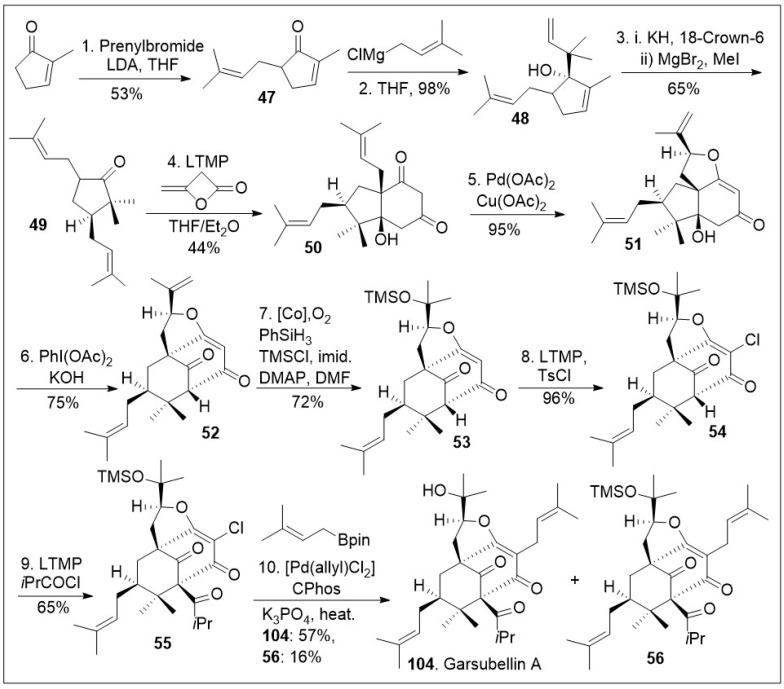
Total synthesis of garsubellin A.

A similar approach has been applied to the caged meroterpenoid garsubellin A (**104, Scheme 6**) [73]. The total synthesis was initiated with methylcyclopenenone, which was α-prenylated to generate compound **47**, followed by 1,2 Grignard reaction to access allylic alcohol **48**. The resultant compound **48** was subjected to an anionic oxy-Cope rearrangement to provide bisprenylated cyclopentanone **49**. Then, compound **49** underwent an annulation reaction with β-lactone diketene to produce diketone **50**, followed by Pd(OAc)_2_/Cu(OAc)_2_ mediated unusual Wacker-type-intramolecular cyclization to provide tricycle system **51**. Tricyclic system **51** was treated with PhI(OAc)_2_ under basic condition for an oxidative ring expansion leading to compound **52,** the needed bicyclo[3.3.1]nonane ring system that upon cobalt catalyzed Mukaiyama hydration reaction provided tricycle **53**. At this point in the synthesis compound **53** was treated with lithium tetramethylpiperidide (LTMP), and TsCl to introduce the α-vinylogous chlorine to generate compound **54**, followed by acylation to introduce the congested quaternary center leading to compound **55**. Various types of alkylation reactions are feasible for such compound, but alkylation with borane prenyl system mediated by [Pd(allyl)Cl_2_], and catalytic CPhos ligand under basic conditions introduce the prenyl group and remove the silyl protecting group to provide garsubellin A (**104**), as well as compound **56**, which could be treated with acid or base to yield garsubellin A (**104**).

The polycyclic fused ring meroterpenes such as berkeleyone A have displayed anti-inflammatory activity [74], while preandiloid A has shown antimicrobial properties. Asperterpene A was identified as a potent BACE-1 inhibitor with an IC_50_ value of 78 nM against this enzyme, out preforming commercial BACE-1 inhibitors. Furthermore, in vivo animal model studies were congruent with the in vitro efficacy studies of this natural product [74]. These findings indicate that these compounds require further studies and synthetic strategies to access them in large quantities.

**Scheme 7:**
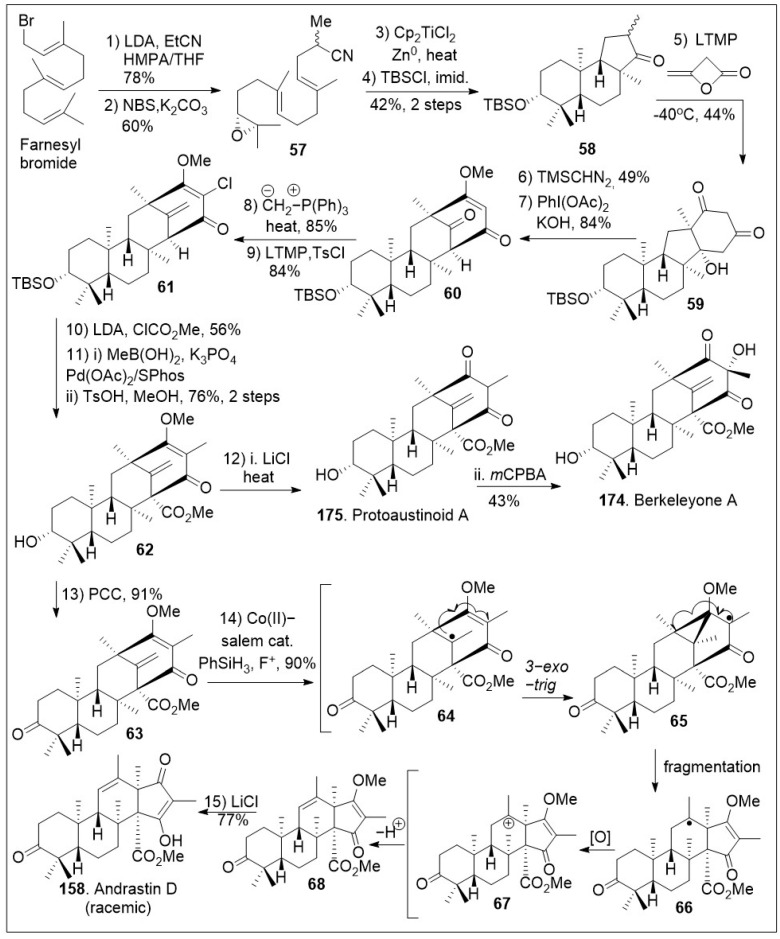
Synthesis of protoaustinoid A, berkeleyone A and andrastin D.

A successful approach to these natural products involves the use of a cyclization reaction to set up the tricyclic system in a single step as shown in Scheme 7 [73]. The synthesis began with farnesyl bromide being subjected to alkylation with the anion of propionitrile, followed by one-pot bromohydrin formation/cyclization reaction sequence to generate epoxide **57**, which upon reductive epoxide-opening radical cyclization furnished tricyclic ketone **58**. Then, ketone intermediate **58** was subjected to annulation reaction with β-lactone diketene to produce diketone **59**, which was O-methylated, followed by oxidative ring expansion mediated by PhI(OAc)_2_ under basic conditions to furnish bicyclo[3.3.1]nonane ring system **60**. The newly formed tetracyclic **60** was exposed to Wittig olefination and subsequent chlorination reaction to yield halogenated polycyclic compound **61**.

**Scheme 8:**
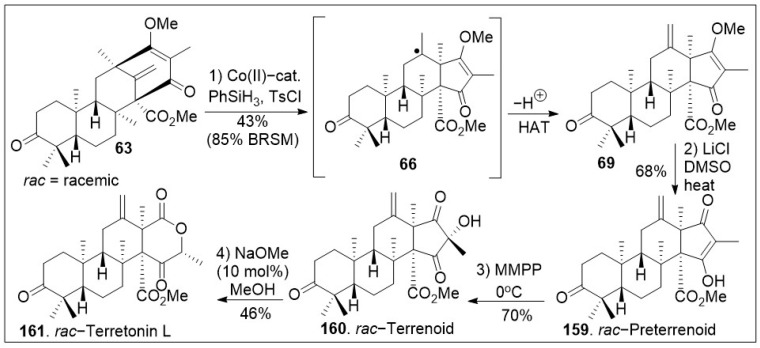
Synthesis of preterrenoid, terrenoid, and terretonin L natural products.

Upon further three-step α-alkylative methyl ester functionalization, methylation via Suzuki coupling reaction of the vinyl chloride with MeB(OH)_2_, and acid mediated deprotection yielded advanced intermediate **62**. Then, compound **62** was subjected to the final chloride-mediated demethylation, leading to the DMOA-derived metabolite, protoaustinoid A (**175**). Compound **175** was further oxidized with *m*CPBA to provide berkeleyone A (**174**). In addition, advanced polycyclic compound **62** was treated with pyridinium chlorochromate (PCC) to generate compound **63**, which was subjected to Shigehisa’s cobalt(II)-catalyzed hydroalkoxylation reaction leading to polycyclic intermediate **68**. Finally, this compound was treated with LiCl to free the trapped enolate system, yielding the natural product andrastin D (**158**) in racemic form. Furthermore, as shown in Scheme 8, the tetracyclic compound **63** was subjected to Carreira’s alkene hydrochlorination conditions to provide the polycyclic compound **69** via hydrogen-atom-transfer (HAT) process involving radical intermediate **66**. Subsequent demethylation of polycyclic compound **69** mediated by LiCl yielded the natural product preterrenoid (**159**), which underwent stereoselective α-oxidation mediated by magnesium monoperoxyphthalate (MMPP) to afford terrenoid (**160**). Upon ring-expansion via retro-Claisen/esterification cascade reaction under basic conditions, compound **160** was converted to racemic terretonin L (**161**). This synthetic strategy is efficient at enabling the generation of multiple members of this meroterpene family in good overall chemical yields [73]. This approach is not asymmetric, however, there are multiple opportunities to introduce asymmetry.

**Scheme 9:**
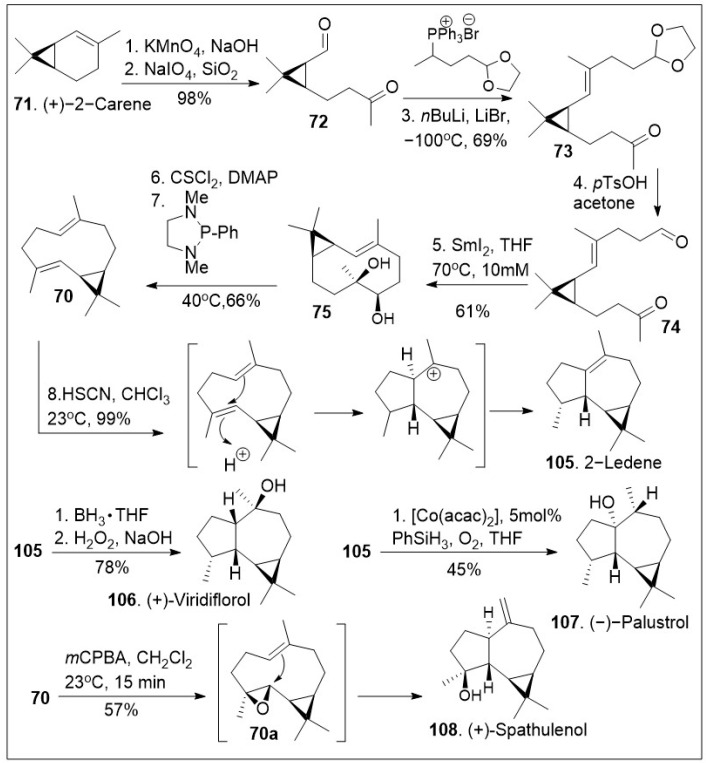
Biomimetic synthesis of several aromadendrene terpenoids.

Forging complicated carbon-carbon bonds in complex natural products represents a challenge that requires groundbreaking synthetic endeavors or engaging in biomimetic synthesis, the process of imitating nature’s way to make molecules. Biomimetic synthesis of the psiguadial meroterpenoids featuring the key cationic cyclization reaction using terpene (+)-bicyclogermacrene **70** as the platform has been reported [75] and it is depicted in Scheme 9. The synthesis commenced with the generation of ketone-aldehyde **72**, which was generated from (+)-2-carene (**71**) upon treatment with KMnO_4_ to mediate the dihydroxylation, and subsequent oxidative diol cleavage reaction. Compound **72** was further treated with Wittig reagent **A** under basic reaction conditions to provide compound **73**, which was deprotected, and cyclized under samarium mediation to provide the 10-membered cyclic system **75**, which was then converted to (+)-bicyclogermacrene **70** with the required *E*-olefin geometry using a modified Corey-Winter protocol. Compound **70** was then converted to 2-ledene (**105**) via acid facilitated intramolecular cationic cyclization. The natural product **105** was transformed to (+)-viridiflorol (**106**) upon treatment with hydroboration/hydroxylation conditions. Alternatively, compound **105** was subjected to Mukaiyama’s cobalt-catalyzed phenylsilane/O_2_ hydration conditions to provide natural product (−)-palustrol **107**. Finally, epoxidation of compound **70** was facilitated upon treatment with *m*CPBA, followed by intramolecular cyclization provided (+)-spathulenol (**108**). 

**Scheme 10:**
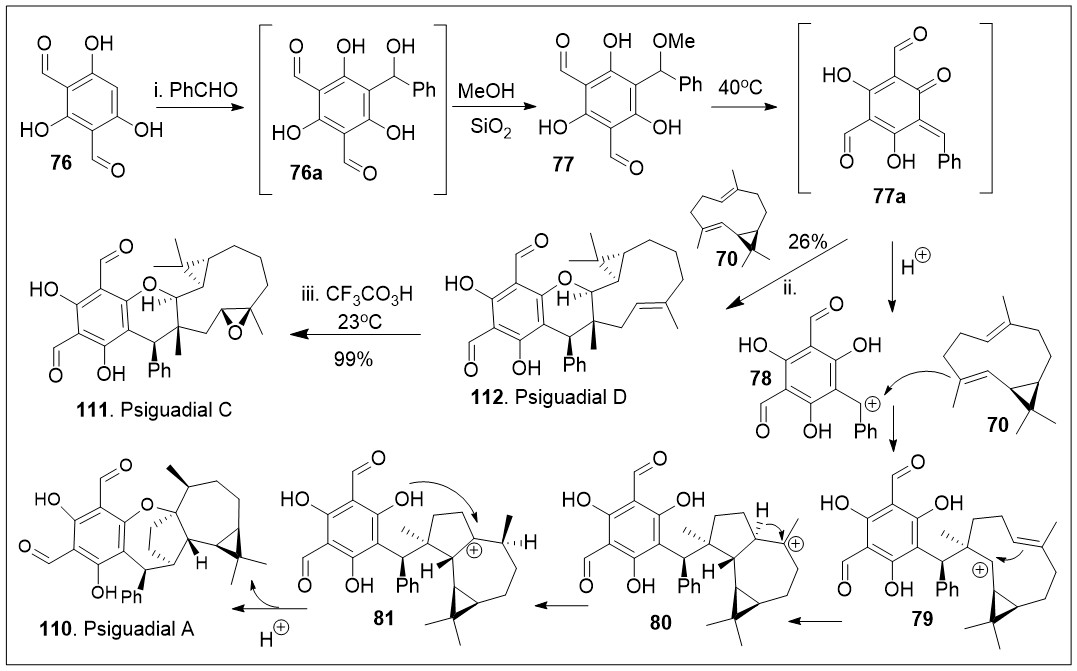
Biomimetic synthesis of psiguadial A and C.

As highlighted in Scheme 10, biomimetic approaches allow for flexible synthesis of various members of the same natural product family [75]. Thus, compound **70** was treated with intermediate *o*-QM **77** to enable the hetero-Diels-Alder (HDA) reaction, in order to yield the natural product psiguadial D (**112**). The compound *o*-QM **77** was synthesized in situ as the Knoevenagel-type condensation reaction product between bisformylated phloroglucinol **76** and phenyl aldehyde. Furthermore, **112** was converted to psigudial C (**111**) via epoxidation reaction. Under the similar reaction conditions, the *o*-QM **77** was unified with compound **4** via sequential cationic intermolecular coupling reaction followed by intramolecular cyclization cascade reactions. Briefly, intermediate **77** under acidic conditions generated the benzylic carbocation, which was able to react with compound **70** via addition reaction to yield intermediate **79**. Compound **79** underwent an intramolecular cyclization, and a 1,2-hydride shift to establish the most stable cation **81**. Finally, intramolecular cyclization from the phenolic system facilitated the installation of the last 7-member ring system of the natural product psiguadial A (**110**).

**Scheme 11:**
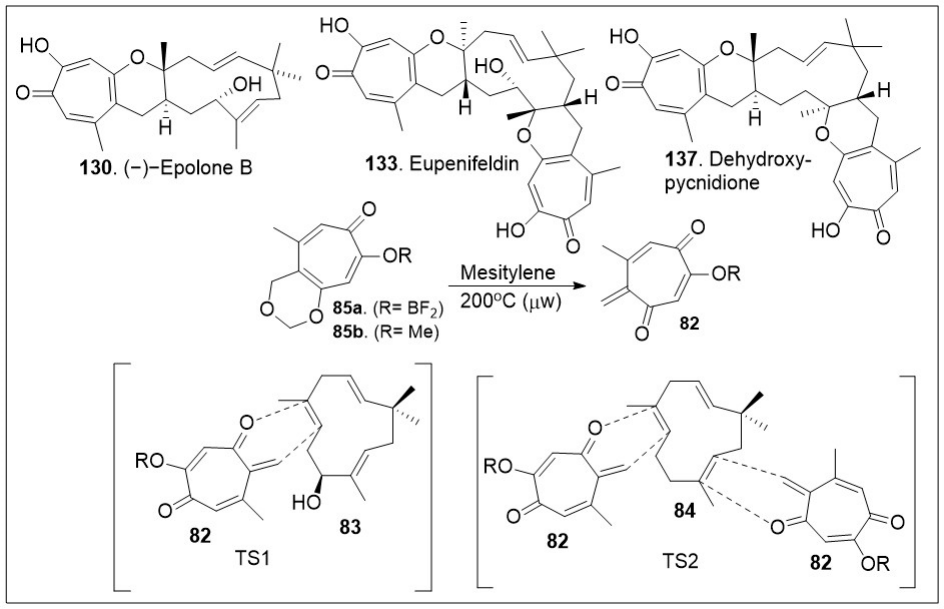
Hetero-Diels–Alder (HDA) strategy to access the tropolones.

The sesquiterpene-tropolones belong to a class of structurally distinctive meroterpenoids characterized as sesquiterpene mono- and bistropolones, which includes pycnidione and eupenifeldin, featuring a unique polycyclic ring system with rich stereochemistry. These compounds are isolated from *Neosetophoma* sp. a species of fungi. These compounds were tested against a series of cancer cell lines: human breast cancer (MDA-MB-231), two human ovarian cancer cell lines (OVCAR-3 and OVCAR-8), human mesothelioma (MSTO-211H), and human lung cancer (A549) to determine their IC_50_ values; the most potent compound, eupenifeldin (**133**, Scheme 11) had IC_50_ values of 2.83, 0.33, 0.02, 0.08, 1.33 μM for the above listed cell lines respectively [76]. To further evaluate the cytotoxicity of these compounds towards healthy tissues a mitochondrial toxicity assay was conducted for compounds **129, 131** and **133** (Figure 5). The results of this experiment showed no cytotoxicity up until a maximum dose of 12.5 μM, which was well above the majority of the IC_50_ values for the compounds tested [76]. Further study of this natural product was conducted by Maldonado et al. focusing on its effects on a panel of ovarian cancer cell lines. The antiproliferative effect (EC_50_) of eupeninfeldin on three ovarian cancer cell lines (OVCAR-3, OVCAR-5, OVCAR-8) and nontumorigenic fallopian tube cells (FTT3-Tag) were determined to be 10, 11, 12, and 170 nM respectively [77]. The results indicate the selectivity of the compound for cancer cell models over normal tissue with a greater than 10-fold difference. Additional mechanistic studies included cell migration assays, which provided favorable outcomes [77]. To validate their in vitro findings, hollow fiber studies were conducted in murine models. OVCAR-3 and OVCAR-8 hollow fibers were implanted in mice, which were treated with eupeninfeldin or vehicle daily for 4 days. A significant reduction of OVCAR-3 cell model was observed while no significant inhibition of OVCAR-8 cell model was recorded suggesting the compound is working via a specific pathway. To further investigate the mechanism of action specific protein evaluation was conducted for apoptosis pathway (caspase 3/7 activation, PARP cleavage, autophagy assays) and global protein analysis using tandem mass tag (TMT), a chemical label technique that facilitates sample multiplexing in mass spectrometry (MS)-based quantification and identification of the proteome. Bafilomycin A1, a member of macrolide antibiotics and an autophagy inhibitor, was used as a co-treatment with eupenifeldin on their selected ovarian cancers cell lines (OVCAR-3, OVCAR-5, and OVCAR-8) to determine if this compound is mediating cell death through autophagy as a primary cell death modality. The resultant data indicated that indeed bafilomycin was able to significantly rescue the cells from death. Their combined findings indicate that this natural product displays selectivity. It is likely mediating cell death through autophagy [77]. Pycnidione (**140**, Figure 5) is another example of bioactive sesquiterpene-bistropolone meroterpenoid natural products. First isolated from fungal species, *Phoma* sp., this compound has been studied extensively for its biological activities. Kaneko studied the effects of pycnidione on leukemia cell models [78]. This compound alone or in combination with bleomycin against a panel of human cancer cell lines had a significant anti-proliferative effect. Ongoing studies indicate that pycnidione inhibits of topoisomerase II at an IC_50_ value of 19 μM and modulates the cell cycle at the G2 phase [78]. These biological studies highlight the importance of having access to large quantities of these natural products for further derivatization to improve efficacy. The recent total synthesis of the structurally revised flagship members of the pycnidione family, namely (+)-pycnidione (**140**), dehydroxypycnidione (**137**) and (−)-epolone B (**130**) was elegantly designed on a modular [4+2] inverse electron-demand hetero-Diels-Alder (HDA) reaction between the in situ generated *ortho*-quinone methide (*o*-QM) **82** and the corresponding dienophile, hydroxyhumulene **83** or humulene **84** (Scheme 11). While the majority of [4+2] cycloaddition reactions require heat, microwave assistance can also promote the rection, and minimize decomposition of starting materials or intermediates. A unified synthetic endeavor was recently used to generate various members of these complex meroterpenes through the in situ generation of compound **82** (Scheme 11). The resultant transition state of either dienophile **83** or **84** can proceed either as a single monocyclic formation step, TS1 or a bis-cyclization formation, TS2. They indicated that the reaction can be modified to favor one or various products. However, the diastereomers of eupenifeldin series have yet to be synthesized and remain a highly active area of research [79].

**Scheme 12:**
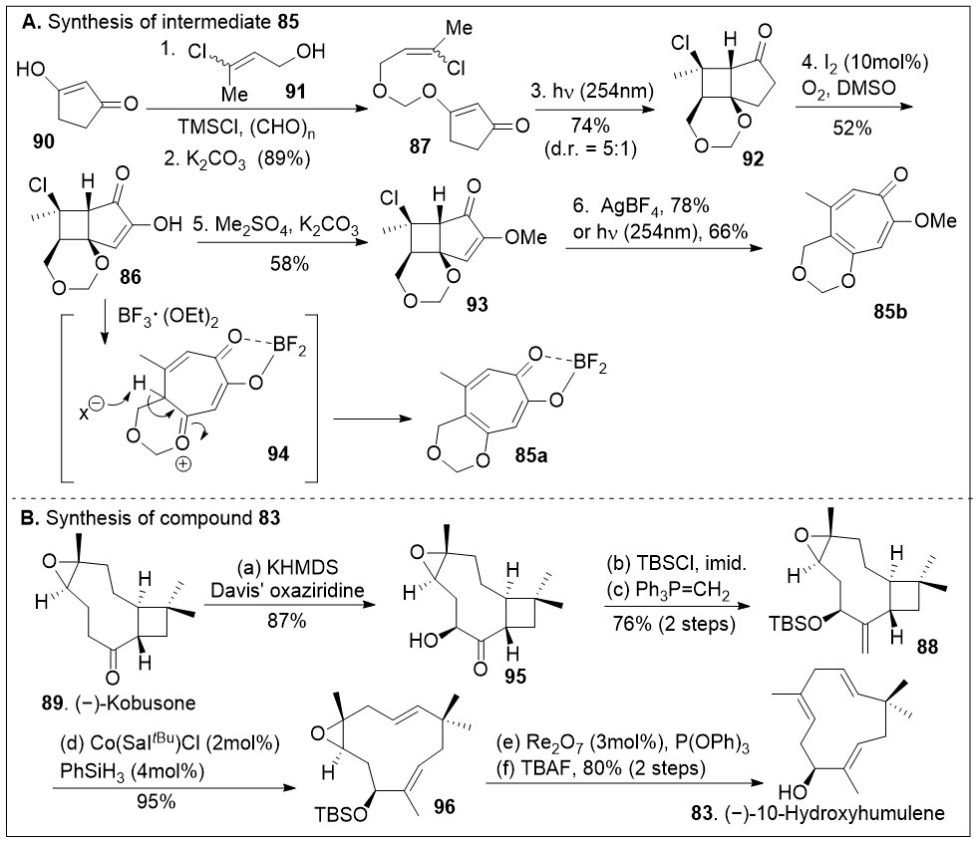
Synthesis of quinone methide precursor and (−)-10-hydroxyhumulene.

The synthesis capitalizes on the advantages to access building blocks **83**, *o*-quinone methide precursors **85a** and **85b** in sufficient quantities (Scheme 12) [79]. Their strategic synthesis began with 1,3-cyclopentanedione **90**, followed by O-alkylation with **91**, which is formed in situ with paraformaldehyde and chlorotrimethylsilane under basic conditions, providing the vinyl chloride **87** that can easily undergo a [2+2] intramolecular photocycloaddition furnishing tricycle **92**. Light-driven [2+2] cycloaddition reactions are the most direct approach to build tetrasubstituted cyclobutanes that may lead to diverse structures. Upon one-pot iodination/Kornblum oxidation, the tricycle **92** was converted to hydroxy enone **86**, followed by O-methylation provide intermediate **93**. Use of either AgBF_4_ or UV light (254 nm) facilitated the ring expansion via fragmentation of compound **93** to provide the methoxy-tropolone **85** in good yields (Scheme 12A). Alternatively, treatment of compound **86** with Lewis acid, boron trifluoride diethyl etherate, resulted in tropolone **84**. Thus, the use of simple transformations easily generates the large ring-containing molecule in sufficient quantities. (−)-Kobusone **89** was converted to the corresponding α-hydroxylated product **95** via Davis’ oxiziridine treatment as shown in Scheme 12B. The resultant α-hydroxyl group was protected with a silyl group, followed by 1-carbon Wittig reaction yielding compound **88**, setting the stage for the ring opening. This reaction previously discovered by the Shenvi group [80] highlights the strategic use of Cobalt/Sal/silane catalyst systems to mediate a Hydrogen-Atom Transfer (HAT) retrocycloisomerization of compound **88** to afford compound **96** in excellent yields. Rhenium catalyzed deoxygenation and silyl group removal with TBAF provided the desired compound **83** as the starting material for the cycloaddition reaction.

**Scheme 13:**
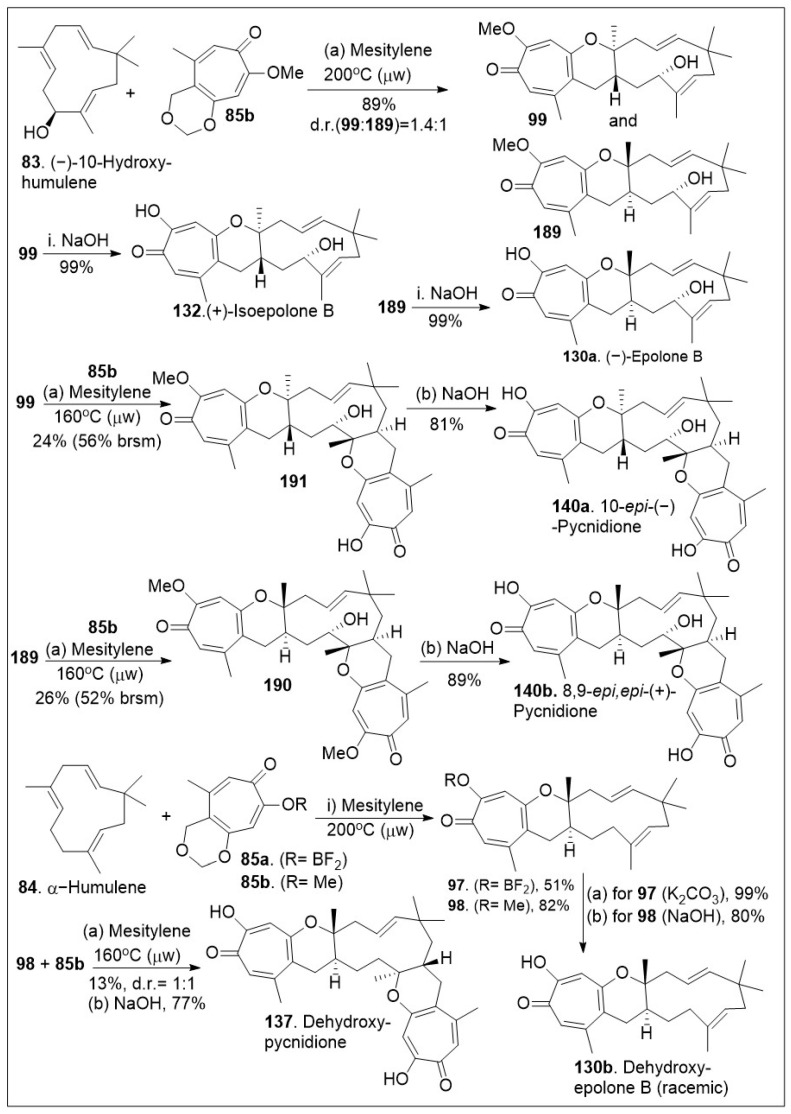
Synthesis of (−)-epolone B and family members.

The application of this HDA reaction to various ene-systems is an efficient approach to generate natural products with high order architecture as shown in Scheme 13. Furthermore, these types of cyclization reactions tend to be selective and can be applied to large scale settings, enabling carbon-carbon bond formations in an economical manner. With the in situ generated *o*-QM **82** from tropolone **85a** or **85b** and dienophile **83**, the platform was established for the [4+2] inverse electron demand HDA reaction [79]. The thermal microwave assisted HDA reaction between (−)-hydroxyhumulene **83** and *o*-QM (generated in situ from precursor **85**) provided cycloadducts **99** and **189**. Note that mesitylene was used rather than toluene to reach higher boiling points and provide the desired products in modest yields. Next deprotection of methoxy group of compounds **99** or **189** via base, led to the natural product epimers **132** or **130a** respectively. Then, compound **99** was subjected to HDA reaction again with compound **85b** to yield **191**, which was deprotected under basic reaction conditions to afford the natural product epimer compound **140a**. The same treatment could be provided to compound **189** to yield the natural product epimer **140b** (8,9-*epi*, *epi*-(−)-pycnidione) in good yields. Finally, the HDA reaction of between humulene **84** (no chirality) and in situ generated *o*-QM (from **85a** or **85b**) was conducted to produce compounds **97** or **98** respectively. Deprotection of these compounds led to the natural product **130b** (dehydroxyepolone B) as a racemate in good yields. Compound **98** was further treated with dienophile for a second HDA reaction under the same reaction conditions to provide the adduct precursor of **137** in poor yields indicating the second HDA reaction is more difficult for **83** than for the allylic system **84**. Perhaps, the presence of the hydroxyl group lowers the barrier slightly in favor of that second reaction. Deprotection of the methoxy group provided natural product **137** [79].

**Scheme 14:**
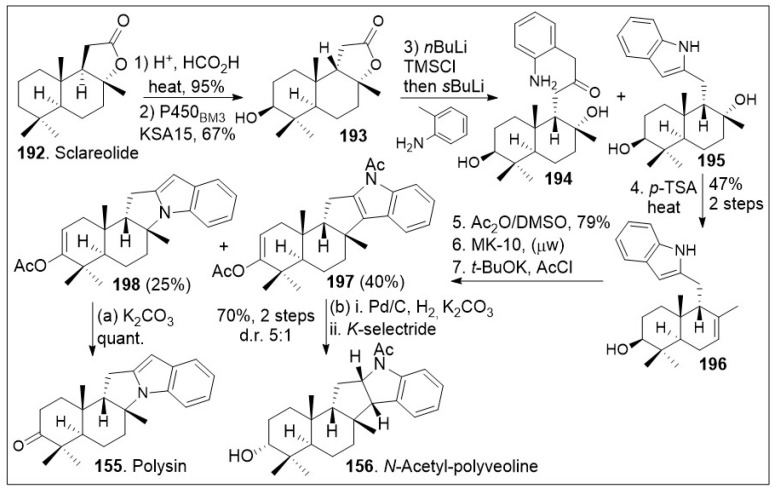
Chemoenzymatic synthesis of drimane meroterpenoid polysin.

**Scheme 15:**
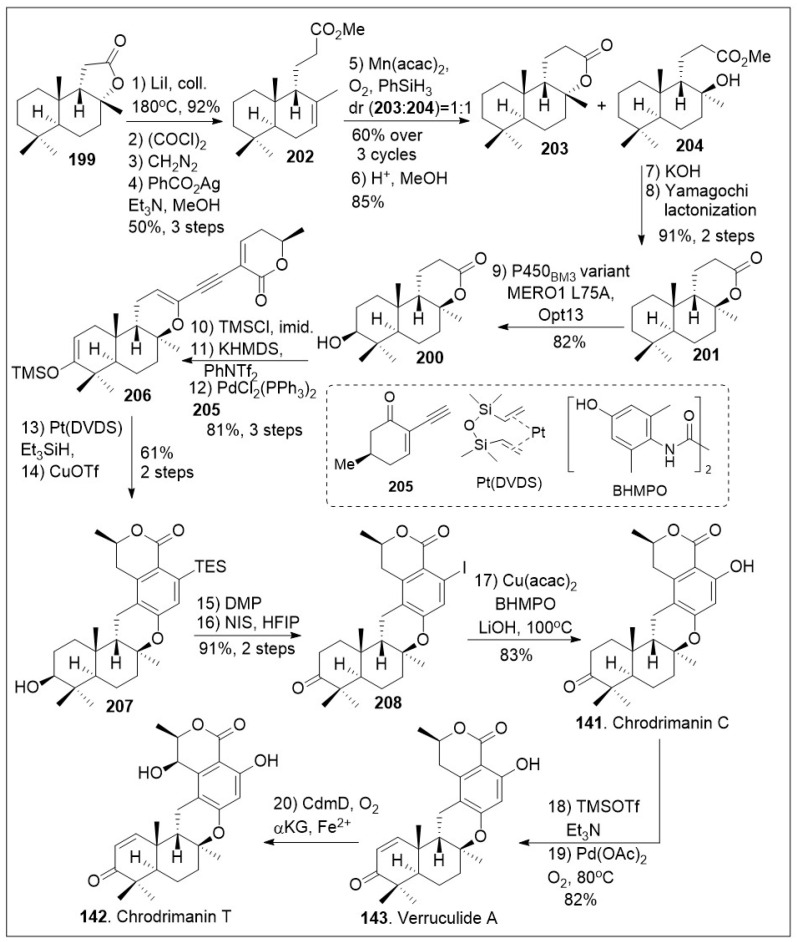
Chemoenzymatic synthesis of verruculide A, chrodrimanin C, and T.

Recent synthetic approaches towards the *trans*-*syn*-fused drimane meroterpenoids have capitalized in advancements in chemoenzymatic reactions [81] to facilitate carbon economic and diversity oriented synthetic strategies. The total synthesis application using a chiral-pool natural product is highlighted in Scheme 14. The synthesis commenced with the commercially and readily available sclareolide **192**, which was treated with acid to epimerize the β-hydrogen, and re-formation of the *syn*-lactone (**199**, Scheme 15). Then, stereoselective enzymatic hydroxylation of the methylene group adjacent to the quaternary center using P450_BM3_ variant KSA15 provided compound **193** in respectful yields in gram scale. Compound **193** was treated with *n*BuLi, trapped with a silyl group, and coupled with 2-methyl aniline anion to provide compounds **194** and **195**. This indole reaction known as Smith-modified Madelung indole synthesis protocol is usually high yielding and relatively compatible with other functional groups. This mixture was converted to **196** under acid treatment to facilitate the dehydration. System **196** was then exposed to Albright-Goldman oxidation to generate the corresponding ketone, which was further subjected to two-step microwave-promoted intramolecular Friedel-Crafts cyclization reaction and subsequent in situ trapping of the enolate with acetic anhydride, affording enol ether adducts **197** and **198**. Next palladium catalyzed hydrogenation and subsequent K-selectride mediated diastereoselective reduction of enol ether adduct **197**, provided *N*-acetyl-polyveoline (**156**). In parallel, compound **198** was treated with potassium carbonate to release the acetate group, resulting in the natural product polysin (**155**) in good yield. 

To generate other meroterpenoid family members (Scheme 15), lactone **199** was converted to methyl ester **202** through a series of four linear steps [81]. The lactone was first opened using heat, followed by three-step Arndt-Eistert homologation to furnish the corresponding methyl ester **202** in excellent yields. Then, compound **202** was treated with Mukaiyama hydration protocol which led to a mixture of lactone **203** and tertiary alcohol **204** in relatively good yields. In addition, lactone **203** was converted back to methyl ester **202** and recycled to increase the overall yield. Upon saponification and subsequent Yamaguchi lactonization of tertiary alcohol **204**, the *trans*-*syn*-*trans*-fused lactone **201** was generated. At this point, the application on enzymatic transformation at the methylene group adjacent to quaternary center was conducted. The enzymatic hydroxylation took place under P450_BM3_ variant MERO1 L75A on a preparative scale to produce alcohol **200**. Next vinyl triflate formation was conducted upon treatment of alcohol **200** with silyl group protection and trapping of the enolate to provide the corresponding triflate, which underwent Sonogashira coupling reaction with alkyne **205**, to generate dienyne **206**. Subsequent hydrosilylation of the newly formed dienyne **206** catalyzed by Platinum(0)-1,3-divinyl-1,1,3,3-tetramethyldisiloxane complex known as Pt(DVDS) in the presence of Et_3_SiH, afforded the desired hydrosilylated triene intermediate, which could readily undergo 6π electrocyclization/aromatization reaction under copper(II) triflate to yield arene **207**. The resultant compound was treated with Dess-Martin periodinane (DMP) to mediate the oxidation of the secondary hydroxyl group followed by desilylative iodinization under NIS mediation to provide iodo-arene **208**. Compound **208** was subjected to copper-catalyzed hydroxylation of aryl halides under mild conditions, namely Cu(acac)_2_ in the presence of *N,N*′-bis(4-hydroxyl-2,6-dimethylphenyl)oxalamide (BHMPO) and base to yield chrodrimanin C (**141**). The natural product **141** can be converted to verruculide A (**143**) upon treatment with Saegusa 2-step protocol to install the α,β-unsaturated system. Furthermore, this natural product can be converted to chrodrimanin T (**142**) under Fe(II)/α-ketoglutarate-dependent oxygenase treatment to introduce the hydroxyl group stereoselectively at the γ-position of the lactone ring. Overall, the Renata group provided an impressive use of enzymatic and modern organic chemistry reactions [81]. 

**Scheme 16:**
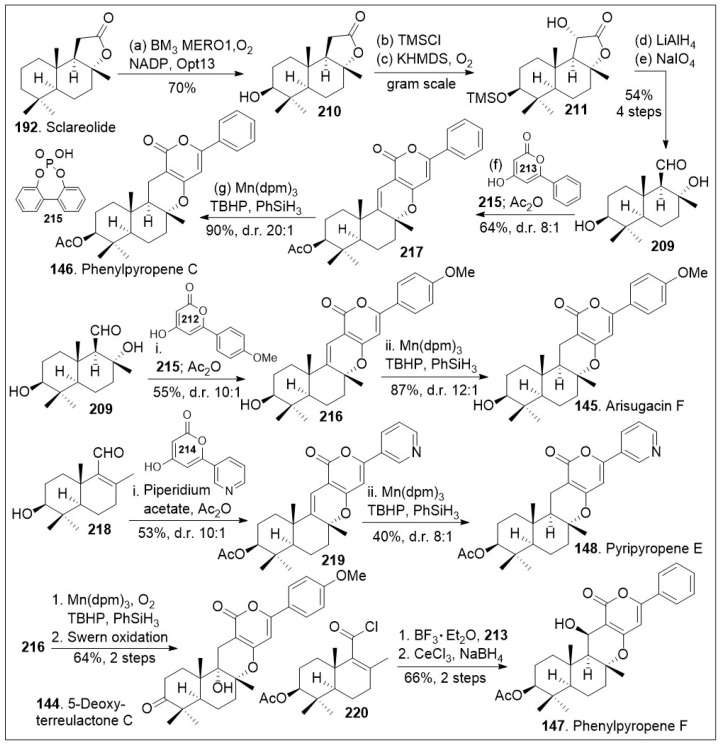
Modular total syntheses to access phenylpyropene C, and related family members.

Another effective synthetic approach using natural products as a chiral pool is the strategy towards the arisugacin natural products. These drimane meroterpenoids have been isolated from *Penicillium* sp. and other sources. Their members have demonstrated acetylcholinesterase inhibitory activity in the nanomolar range [82]. Access to both libraries and large quantities of these compounds is desirable to interrogate their mode of action. Stypodiol is a pentacyclic meroterpenoid isolated from the brown algae *S. flabelliforme*, which displays a broad range of biological activity. Stypodiol has shown anti-proliferative effects against a number of cancer cell lines and also has antimicrobial capacity. This compound showed promising anti-proliferative activity in the low micromolar range against a human neuroblastoma cell line (SH-SY5Y) [83]. While multiple cancer cell lines were tested most of them showed weak anti-proliferative effects, indicating the compound is likely to have selectivity towards a specific cancer subtype. Therefore, efficient synthetic approaches to this family of compounds are desirable. A recent rapid and modular approach using modern organic chemistry, such as radical-based reactions combined with chemoenzymatic reactions has been reported [81]. As shown in Scheme 16, the synthesis commenced with natural product sclareolide **192**, which underwent enzymatic hydroxylation using P450_BM3_ variant BM3 MERO1 in the presence of thermostabilized phosphate dehydrogenase (Opt13) at the methylene group adjacent to the quaternary center. The corresponding compound **210** was protected with a silyl group followed by stereoselective α-hydroxylation, conducted via trapping of the enolate with oxygen to efficiently generate compound **211**. Next, lactone **211** was reduced to the triol system followed by oxidative cleavage of the diol moiety, rapidly furnishing the key aldehyde **209**. Aldehyde **209** was coupled with pyrone **213** via a formal [3+3] cycloaddition/hydrogen atom transfer (HAT) reaction mediated by phosphoric acid **215** to generate cyclized product **217**, which was acetylated in a one-pot procedure with acetic anhydride. Phosphoric acid catalyst promoted in situ alcohol dehydration and formal [3+3] cycloaddition to provide the shown stereocenter as the major product. Then, it was treated with Mn(dpm)_3_ in the presence of TBHP (*tert*-butyl hydrogen peroxide) and PhSiH_3_ to facilitate a chemoselective HAT reduction reaction to provide the natural product phenylpyropene C (**146**). This Mukaiyama reduction protocol has high stereospecificity as it favored the hydride addition from the bottom face, yielding the product in a 20 to 1 ratio with respect to its diastereoisomer. 

Alternatively, aldehyde **209** was coupled with pyrone **212** via a formal [3+3] cycloaddition reaction mediated by phosphoric acid **215** to generate cyclized product **216**, which was converted to arisugacin F (**145**) via chemoselective HAT reduction reaction mediated by Mn(dpm)_3_ in the presence of TBHP and PhSiH_3_. Compound **209** was dehydrated to provided enal **218**, which was coupled with the shown pyrone via formal [3+3] cycloaddition mediated by piperidium acetate to generate compound **219**. The corresponding compound can be then converted to the natural product pyripyropene E (**148**) via chemoselective HAT reduction reaction using their established reaction conditions for these systems in modest yields and good diastereomeric ratio. In addition, compound **216** was treated with Mukaiyama hydration chemoselective HAT reaction mediated by Mn(dpm)_3_ in the presence of oxygen gas and PhSiH_3_, followed by Swern oxidation to provide 5-deoxyterreulactone C (**144**) in good yields. Finally, the acyl chloride **220** that arises from aldehyde **209** was subjected to Fredel-Crafts acylation reaction with pyrone **213** mediated by BF_3_ etherate followed by cyclization reaction. Then, reduction of the ketone system under Luche reduction conditions provided the natural product phenylpyropene F (**147**). The total syntheses of various members of this drimane meroterpenoid family was achieved through the creative use of strategic enzymatic and chemoselective reactions to provide flexible, modular, and a generalized synthetic route that may facilitate biological studies of these natural products and their derivatives.

**Scheme 17:**
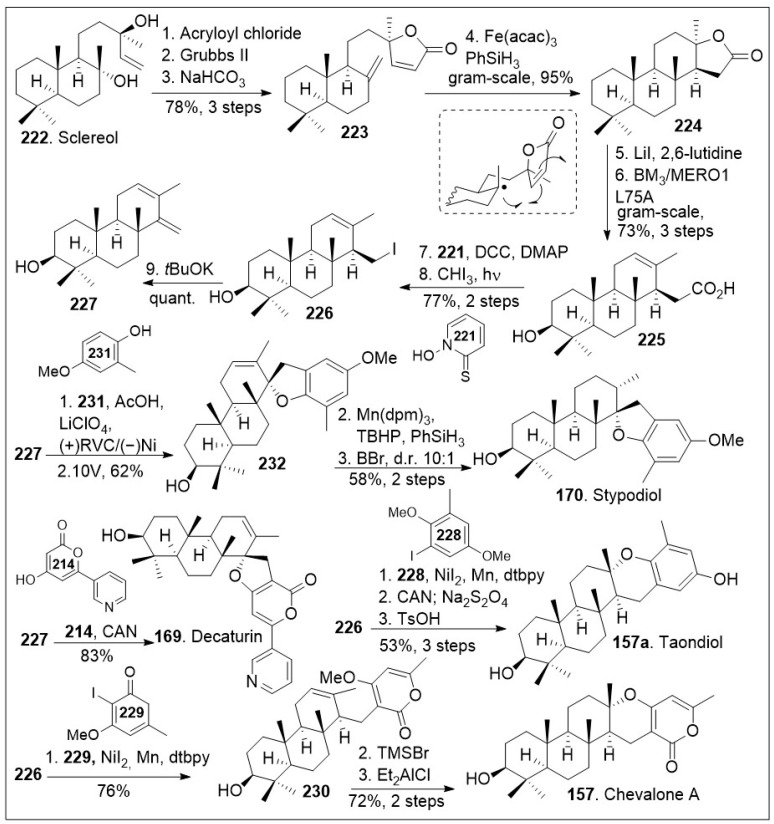
Total Syntheses of stypodiol and chevalone A.

Another modular synthetic effort is highlighted in Scheme 17 to access these natural products, but mainly to stypodiol [84]. The synthesis commenced with the available natural product sclareol **222**, which was converted to intermediate **223** via a 3-step sequential protocol: acylation with acryloyl chloride, γ-lactone ring formation via intramolecular olefin metathesis mediated by Grubbs second generation catalyst, and finally dehydration reaction mediated by base treatment. 

Next, gram-scale HAT-based intramolecular Giese type coupling reaction was conducted on compound **223** with Fe(acac)_3_ in the presence of PhSiH_3_, which goes through the radical transition state shown to preferentially provide the tetracyclic compound **224**. Then, a two-step sequence mediated by lithium iodide resulted in the opening of the lactone ring and generation of the corresponding carboxylic acid. This was subjected to enzymatic hydroxylation using P450_BM3_ variant BM3 L75A, leading to regioselective hydroxylated compound **225**. The resultant compound could be treated with Barton reaction conditions to undergo decarboxylation, followed by photocatalyzed halogenation reaction to generate the corresponding iodide **226**, which was then converted to diene **227** under basic conditions. Then, electrochemical SET-based [3+2] coupling reaction of diene **227** with phenol **231** via constant potential electrolysis (using reticulated vitreous carbon as cathode and Ni as the anode) generated advanced intermediate **232**. Compound **232** was treated with Mn(dpm)_3_ in the presence of TBHP and PhSiH_3_ to induce diastereoselective HAT reduction followed by deprotection to generate the natural product stypodiol (**170**) in good yield. Using a similar synthetic protocol, diene **227** was coupled with excess pyrone **214** via single-electron-transfer (SET) to promote a formal [3+2] cycloaddition mediated by cerium ammonium nitrate (CAN) to yield the natural product decaturin (**169**). The iodide intermediate **226** was subjected to nickel-catalyzed cross-coupling with arene iodide **228**, followed by the redox maneuver mediated by CAN and subsequent acid catalyzed intramolecular cyclization provided natural product taondiol (**157a**). Furthermore, iodide intermediate **226** was subjected to nickel-catalyzed cross-coupling with pyrone iodide **229** to afford compound **230**, which upon Lewis acid catalyzed intramolecular cyclization afforded the desired natural product chevalone A (**157**) in good yields [84]. This synthetic strategy offers the opportunity to access many derivatives of these meroterpene natural product derivatives by fine-tuning the coupling partner. 

**Scheme 18:**
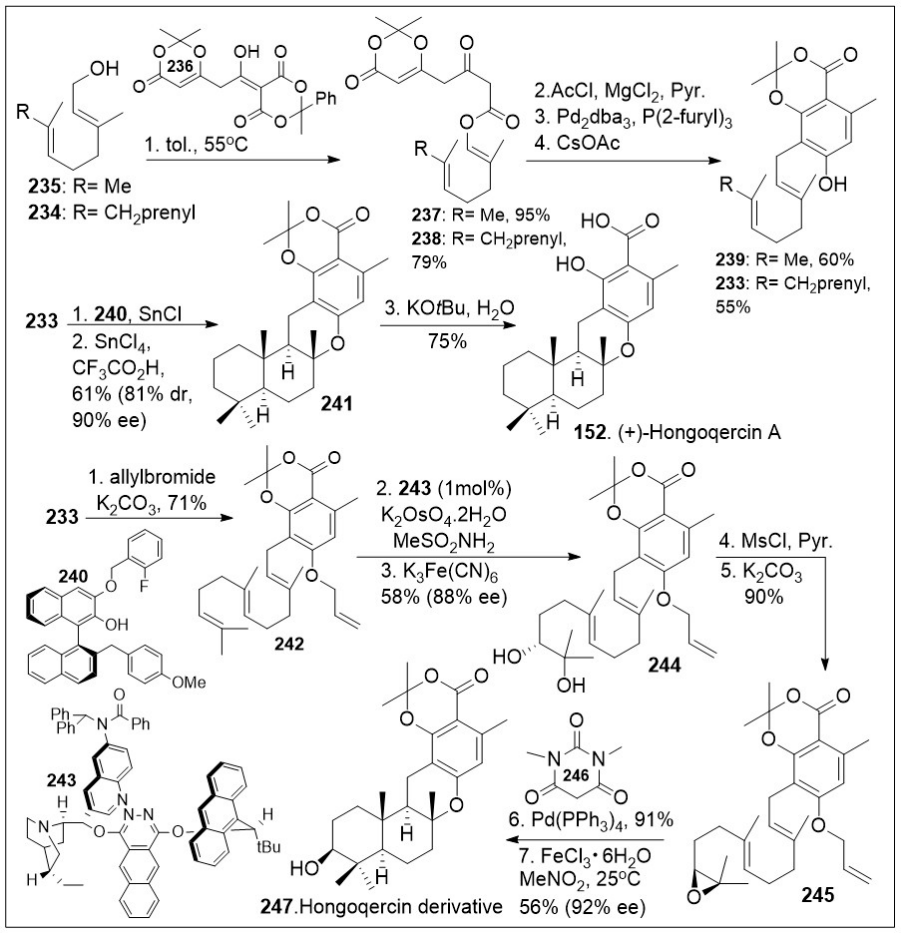
Total synthesis of (+)-Hongoqercin A.

**Scheme 19:**
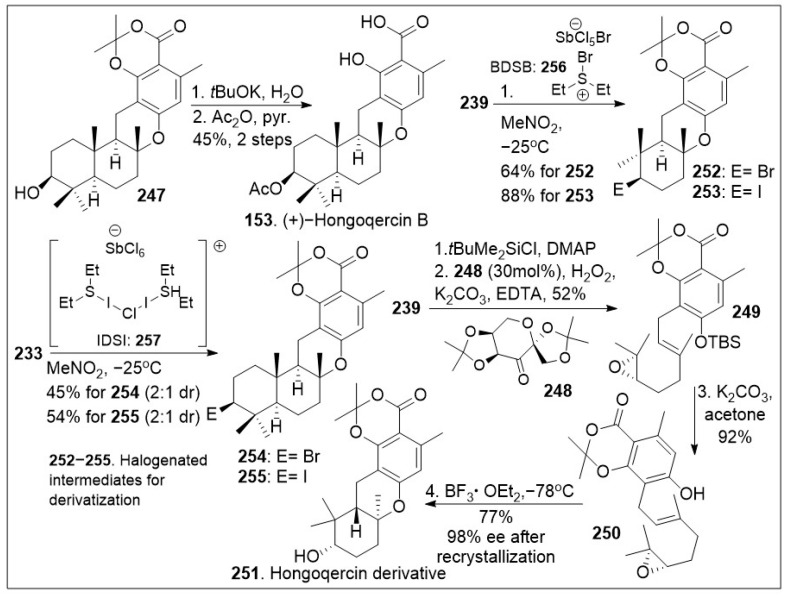
Total synthesis of (+)-Hongoqercin B and halogenated derivatives.

The hongoquercins are tetracyclic meroterpenoid natural products with the *trans-transoid* decalin-dihydrobenzopyran ring system, which display a range of promising bioactivities. This subgroup of the meroterpenoids natural products incorporates a sesquiterpene unit to form the unique hongoquercins. These natural products have attracted attention because they pose a synthetic challenge with four continuous stereocenters and a highly substituted arene scaffold. Access to a modular synthesis can enable the establishment of a medicinal chemistry platform to investigate their exact mode of action. Over the past 25 years multiple synthetic endeavors have been reported towards meroterpenoid (+)-hongoquercin A (**152**, Scheme 18) and (+)-hongoquercin B (**153**, Scheme 19), but a recent simplified approach was disclosed by the Barrett group [85], featuring a sequential polyketide aromatization and cationic polyene cyclization reaction. The synthesis commenced with thermolysis of dioxane-4,6-dione dioxanone **236** in the presence of *trans*, *trans*-farnesyl **234** or geraniol **235** to provide dioxinone β-keto ester **238** or **237** respectively, which upon C-acylation generated the corresponding ester systems. The resultant products underwent highly regioselective decarboxylative allylic rearrangement mediated by Pd_2_dba_3_, followed by in situ aromatization to provide farnesyl resorcylate **233** or the geranyl substituted analogue **239** respectively. Compound **233** was then treated with SnCl_4_ complex with ligand **240** as a dual Bronsted and Lewis acid, in the presence of SnCl_4_ and trifluoroacetic acid, which facilitated the enantioselective protonation of the substrate, followed by facile cationic intramolecular polyene cyclization reaction, leading to meroterpenoid **241**. Compound **241** upon saponification provided the natural product (+)-hongoquercin A (**152**). Alternatively, O-allylation of farnesyl resorcylate **233** with allylbromide under basic condition provided allyl ether **242**, which upon asymmetric dihydroxylation generated diol **244**. This step is followed by mesylating the secondary hydroxyl group, which enables the epoxide formation under a classic S_N_2 reaction to provide epoxide **245**. Compound **245** was then deprotected, followed by biomimetic cationic cyclization of epoxide mediated by Lewis acid, Iron (III) to generate the advanced meroterpenoid derivative **247** in good enantioselective excess. 

As shown in Scheme 19, compound **247** was deprotected to yield the corresponding acid, followed by selective acetylation of the secondary hydroxyl group to provide natural product **153**. Furthermore, halocyclization of farnesyl resorcylate **233** or geranyl-substituted analogue **239** (Scheme 18) in the presence of halogenating reagents **256** (Bromodiethylsulfonium bromopentachloroantimonate V) or **257** (iodonium reagent), provided halogenated meroterpenoid **254**/**255**, or **252**/**253** respectively in good yields [85]. These important intermediates will allow the generation of several derivatives that can further provide insight into the biological mode of action of these natural products. An interesting meroterpenoid derivative of hongoquercin was generated geranyl-substituted resorcylate **239**. This was protected with a silyl group followed by regioselective asymmetric epoxidation with Shi chiral ketone to generate epoxide **249**. The resultant system was deprotected under basic conditions to yield epoxide **250**, which underwent cationic polyene cyclization reaction mediated by boron trifluoride etherate to provide the advanced meroterpenoid derivative **251**. This compound should serve as a chemical tool to interrogate the biological properties of these natural products.

**Scheme 20:**
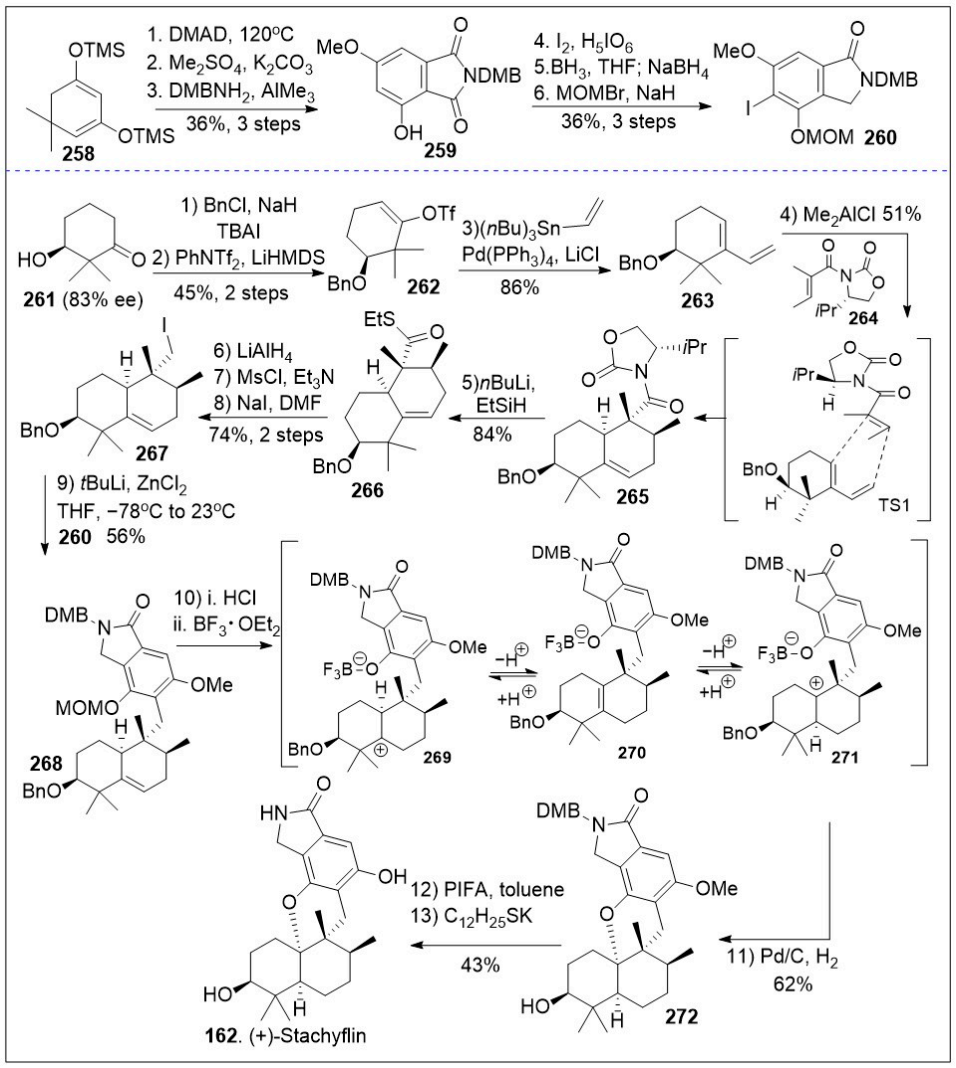
Modular synthesis of tetracyclic meroterpenoids.

A modular synthetic approach to the tetracyclic meroterpenoids was recently reported [86] and it is as illustrated in Scheme 20. The synthesis featuring sterically demanding sp^2^-sp^3^ cross-coupling and acid mediated cyclization/isomerization for installation of the required *cis* or *trans*-decalin stereochemistry of the tetracyclic meroterpenoid scaffold is highlighted. The total synthesis commenced with a solvent-free Alder-Rickert reaction between the dimedone-derived bis-trimethylsilyl enol ether **258** and dimethyl 2-butynedioate (DMAD) provided resorcinol, followed by mono-methylation yielded phenol, which upon thermal treatment in the presence of 3,4-dimethoxybenzylamine (DMBNH_2_) yielded imide **259**. Regioselective halogenation of imide **259** generated intermediate **259a**, which upon regioselective imide reduction provided key isoindolinone **260**. Next diene **263** was generated from β-hydroxyketone **261** via three step protocol, involving the formation of benzyl-protected ketone, subsequent vinyltriflate formation, followed by Stille coupling with vinyl(*n*Bu_3_)Sn mediated by Pd(PPh_3_)_4_. Then, a Lewis acid catalyzed Diels-Alder [4+2] cycloaddition reaction between diene **263** and dienophile **264** provided intermediate **265** as the major product due to the shown transition state. Compound **265**, following a two-step sequential reduction reaction, generated alcohol via thioester intermediate **266**, and it was converted to iodide **267** via two linear steps (mesylation of the alcohol followed by S_N_2 reaction). Then, the key step was conducted, namely sp^2^-sp^3^ Negishi coupling of isoindolinone **260** with iodide **267** to provided 5,6-dehydrodecalin intermediate **268**. Next compound **268** underwent deprotection, intramolecular cation trapping of the phenol group, followed by hydrogenation to yield the cyclized product **272**. This established the required meroterpenoid scaffold. Lastly, global deprotection of intermediate **272** provided the natural product (+)-stachyflin (**162**). 

**Scheme 21:**
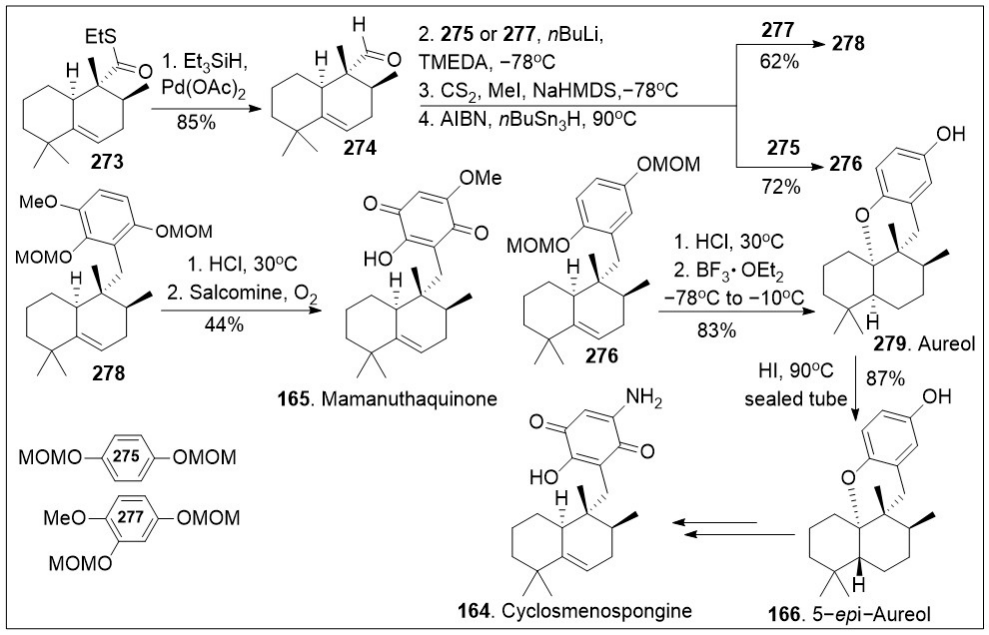
Synthesis of (+)-stachyflin, aureol, cyclosmenospongine, and mamanuthaquinone.

Next as shown in Scheme 21, thioester **273** was reduced to the corresponding aldehyde **274** via Pd(II) in the presence of triethylsilane hydride in good yields. Then, aldehyde was coupled with the anion of arene **275** or **277**, followed by 2-step Barton-McCombie deoxygenation to produce intermediate **276** or **278** respectively. Then, compound **278** was converted to mamanuthaquinone (**165**) via global acid deprotection and salcomine (a coordination complex of salen ligand and cobalt) mediated oxidation reaction sequence in moderate yields. In addition, compound **276** was subjected to deprotection and intramolecular cation trapping with the free phenol group to provide natural product aureol (**279**), which was subsequently converted to 5-*epi*-aureol (**166**) via epimerization of the decalin bridge carbon, using HI. Intermediate **166** is known to generate natural product cyclosmenospongine **164** [86]. The efficient synthesis provides rapid access to various family members and the established platform will enable the generation of derivatives for further biological studies. 

**Scheme 22:**
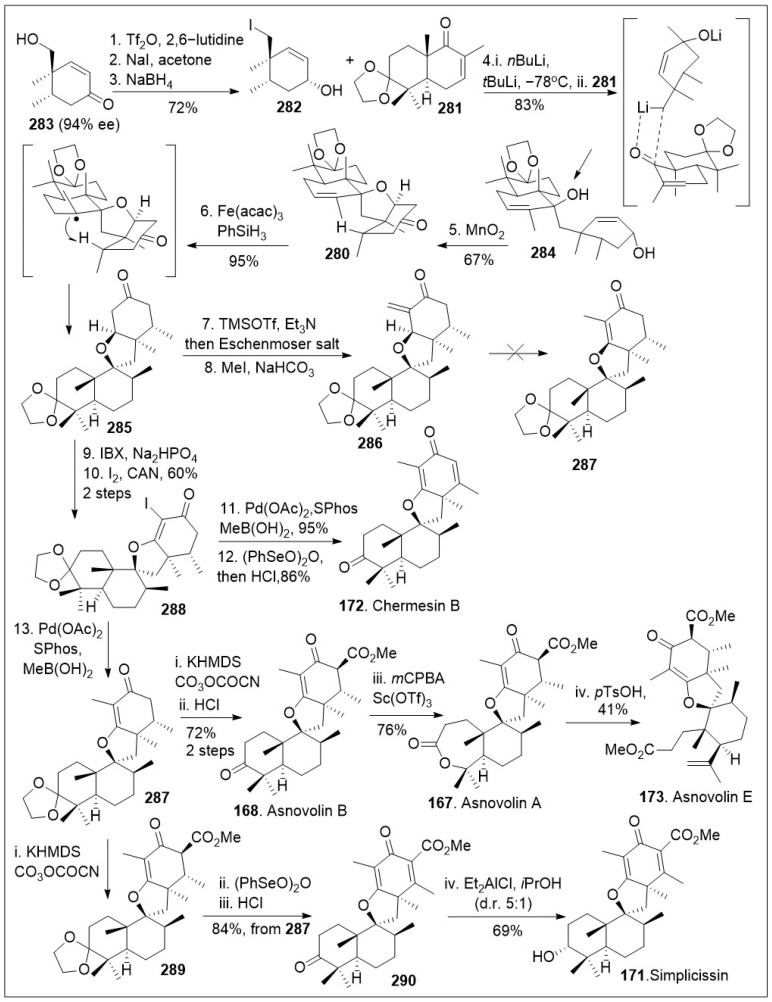
Synthesis of asnovolin A, asnovolin B, asnovolin E, chermesn B and simplicissin.

These unique spiromeroterpenoids share a common biosynthetic pathway that presumably arises from the terpenoid precursor farnesyldiphosphate and display promising biological properties [87]. The total synthesis of asnovolins and related spiromeroterpenoids feature a key lithium mediated coupling reaction to establish the carbon-carbon bond formation that giving rise to the spirocenter, was recently reported by Porco [87], shown in Scheme 22. The synthesis began with known chiral hydroxy enone **283**, which was converted to the corresponding neopentyl iodide via one-pot triflation, followed by an S_N_2 reaction, which upon subsequent Luche reduction, provided allylic alcohol **282**. Coupling reaction between the decalin system **281** and the lithium exchanged form of the iodide **282** via 1,2-addition reaction afforded compound **284**. Next, treatment of the resultant product **284** with MnO_2_ was converted to spirocycle **280** via tandem allylic oxidation, followed by oxa-Michael addition reaction sequence. Spirocycle **280** was then transformed to spirocyclic intermediate **285** via a metal hydride atom transfer (MHAT) process mediated by Fe(acac)_3_ in the presence of hydride source PhSiH_3_, establishing the desired stereochemistry. Enolization of spirocyclic intermediate **285** with TMSOTf followed by treatment with Eschenmoser’s salt, generated exocyclic enone **286**. Under various reactions conditions compound **286** resisted isomerization to the endocyclic enone **287**. Therefore, compound **285** was oxidized with 2-iodoxybenzoic acid (IBX) to the spirocyclic enone system, which was iodinated at the α-carbon to provide the halogenated spirocyclic intermediate **288**. This system was then subjected to Suzuki coupling reaction to generate the desired endocyclic enone **287**, followed by final desaturation, and protecting group removal to generate the spiromeroterpenoid chermesin B (**172**). Compound **288** could be further functionalized via Pd mediated methylation to produce **287** again, which was treated with base to conduct an enolate acylation and deprotection to access the natural product asnovolin B (**168**). The natural product **168** was then subjected to Baeyer-Villiger oxidation in the presence of Sc(OTf)_3_ to yield asnovolin A (**167**). The lactone ring opening of asnovolin A (**167**) was conducted using methanolysis conditions to provide natural product asnovolin E (**173**). In addition, compound **287** was acylated to generate compound **289**, which was desaturated and deprotected to afford advanced spirocyclic intermediate **290**. Finally, compound **290** was subjected to Meerwein-Ponndorf-Verley reduction conditions to produce simplicissin (**171**) as the major product. The powerful linear synthesis enables access to many members of these natural products

**Scheme 23:**
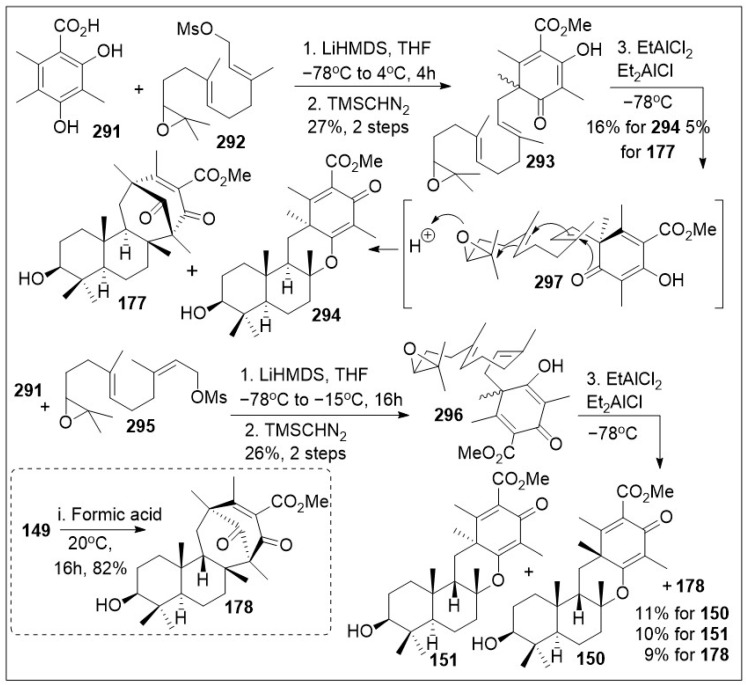
Biomimetic synthesis of asperterpene A derivative and related meroterpenoids.

Asperterpene A and its related family are interesting structures with unique chemistry and biology. Asperterpene meroterpenoids displays an unprecedented 1,2,5-trimethyl-4,9-dioxobicyclo [3.3.1]non-2-ene-3-carboxylic acid moiety that is distinct from other similar meroterpenoids such as berkeleytrione and preaustinoid A. These natural products have exhibited promising BACE1 inhibitory activities in cell-based assays, with IC_50_ values of 60–80 nM. This is more active than LY2811376 (IC_50_ of 260 nM), a potent clinical BACE1 inhibitor produced by Eli Lilly. Additional in vivo studies of one of these natural products showed significant decrease of BACE1 activity and Ab42 levels in 3xTg AD mice, similar to LY2811376 [74]. Therefore, the synthesis of these compounds has clinical relevance, and more potent derivatives could be generated once a streamlined synthetic endeavor has been established. An impressive biomimetic synthesis towards these natural products was recently reported as shown in Scheme 23 [88]. The synthesis began with the coupling reaction of 3,5-dimethylorsellinic acid **291** with (2*E*,6*E*)-10,11-epoxyfarnesyl mesylate **292** as the electrophile under basic condition, followed by in situ methyl esterification to generate (2*E*,6*E*)-10,11-epoxyfarnesyl-5-dimethylorsellinic acid methyl ester **293**. Then, a dearomatization-driven polycyclization reaction was mediated by treatment with alkyl aluminum-pair as a Lewis acid system to promote the intramolecular polyene cyclization cascade reaction to afford asperterpene scaffold **177** and related meroterpenoid **149** via intermediate **297** in a 1 to 3 ratio favoring compound **294**. Alternatively, compound **291** was coupled with electrophile (2*Z*,6*E*)-10,11-epoxyfarnesyl mesylate **295** (the stereoisomer of **292**) using base treatment, followed by in situ methyl esterification to generate compound **296**. The resultant product was treated with alkyl aluminum Lewis acid system to mediate the intramolecular polyene cyclization cascade reaction, which afforded meroterpenoid scaffolds **150**, **151**, and **178**. In addition, it was found that compound **149** could be treated with formic acid to provide natural product **178** in good yields. While the chemical yields are modest, this synthetic approach is remarkable as several centers are established in a single step, generating high complexity rapidly. 

**Scheme 24:**
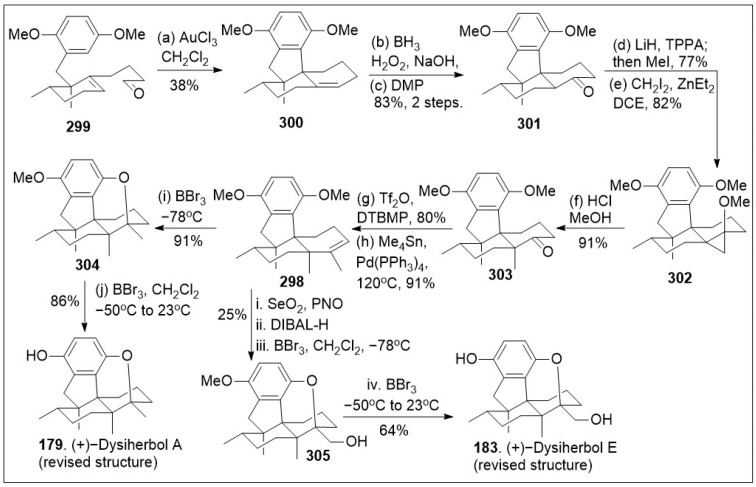
Divergent syntheses of (+)-dysiherbols A and (+)-dysiherbols E.

**Scheme 25:**
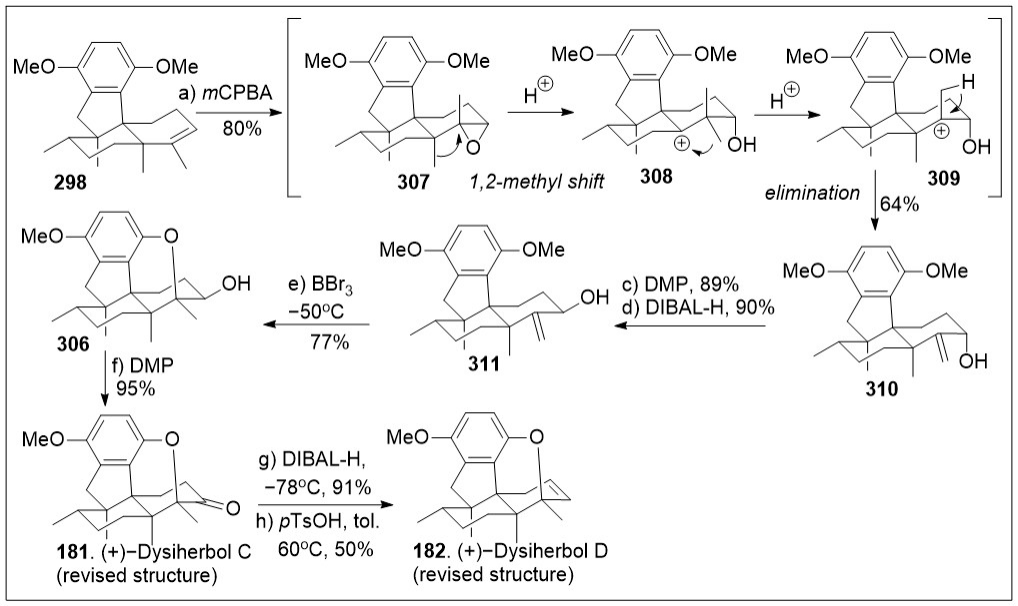
Divergent synthesis of marine meroterpenoids (+)-dysiherbols C and (+)-dysiherbols D.

Dysiherbols A-C are a set of sesquiterpene hydroquinone meroterpenoids first isolated and characterized from *Dysidea* sp., a species of marine sponge found in the South China Sea first isolated by Lin and co-workers [89]. These natural products are structurally unified by a sterically compact 6/6/5/6/6 pentacyclic skeleton embracing an impressive oxabicyclo[3.3.1] ring system. There are 5−6 contiguous stereogenic centers in their molecular scaffold, four of which are quaternary centers. These natural products as extract mixtures were identified to inhibit NF-κB [89]. Isolation and purification of these compounds demonstrated that the pure compounds had potent inhibitory values in the low micromolar range against NF-κB, finding IC_50_ values of 0.49 to 6 μM for dysiherbols A, B, and C respectively. Additionally, these compounds were tested against a human myeloma cancer cell line, NCI H-929, and showed antiproliferation effects in the low micromolar range, with dysiherbols A being the most active with an EC_50_ in the 500 nanomolar range. Recent divergent total syntheses of the revised structures of these sesquiterpene hydroquinone meroterpenoids (+)-dysiherbols A-E (**179**–**183**, Figure 5) was reported as shown in Scheme 24 and Scheme 25 [90]. The modular synthetic strategy began with compound **299** which had been reported to arise from the readily available dimethyl predysiherbol. Compound **299** was treated with gold to catalyze the tandem intramolecular cyclization reactions providing known Schmalz’s intermediate **300**. Hydroboration/oxidative hydroxylation and subsequent Dess Martin Periodinane (DMP) oxidation reaction of compound **300** was converted to ketone **301**. Then, compound **301** was followed by regioselective deprotonation/o-methylation under lithium hydride, α,α,α’-tris(4-hydroxyphenyl)-1-ethyl-4-isopropylbenzene (TPPA) followed by MeI, and subsequent Simmons-Smith cyclopropanation reaction sequence generated cyclopropane **302**, which upon further proteolytic cleavage generated ketone **303**. Then, enol triflate was formed to enable a Stille coupling reaction to afford compound **298**. This compound was subjected to Lewis acid treatment at low temperatures to provide intermediate **304** through an intramolecular oxy-cyclization reaction, which upon increasing of the temperature under the same Lewis acid removed the additional methoxy group to yield the natural product (+)-dysiherbol A (**179**). Alternatively, compound **298** was treated with a 2-step allylic oxidation and reduction reaction sequence, followed by Lewis acid mono deprotection and subsequent intramolecular oxy-cyclization to provide compound **305**. Finally, compound **305** was deprotected to provide natural product (+)-dysherbol E (**183**). 

In parallel, as shown in Scheme 25, compound **298** was treated with *m*CPBA to undergo cationic 1,2-methyl shift and elimination reaction sequence to provide allylic alcohol **310**. The hydroxyl group geometry in **310** was inverted via a 2 step-reaction transformation. Namely, oxidation of the hydroxyl group using DMP treatment, followed by DIBAL-H reduction, which provided allylic alcohol **311**. Then, intramolecular oxy-cyclization of **311** mediated by BBr_3_ generated cyclized ether **306**, which was oxidized with DMP to yield (+)-dysiherbol C (**181**). Reduction of this enone with DIBAL-H, followed by acid mediated dehydration reaction provided (+)-dysiherbol D (**182**). In summary, this synthetic strategy of the tetracyclic core of dysiherbols through the use of critical acid mediated ring construction sequences [90]. This unique synthetic strategy serves as the foundation to access the natural products and their derivatives. 

**Scheme 26:**
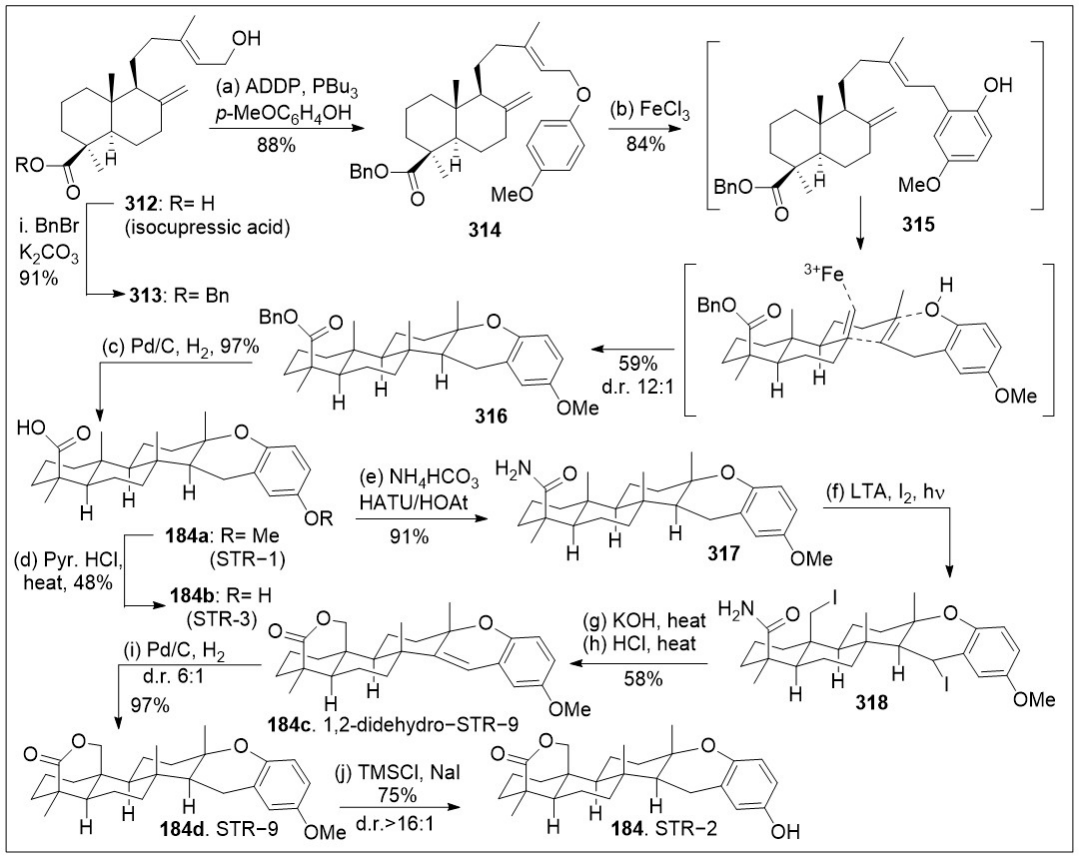
Synthesis of strongylophorine-2 (STR-2).

The strongylophorines meroditerpenoids have been isolated from various sources including the marine sponge *Petrosia corticata*, and they have been demonstrated to be promising proteasome inhibitors [91]. Access to these natural products to develop compound libraries is desirable. A recent streamlined total synthesis of strongylophorine-2 (STR-2, **184**) is highlighted in Scheme 26 [92]. The synthesis capitalizes on a novel iron (III)-mediated rearrangement-cyclization cascade reaction. The synthesis commenced with the esterification of natural product isocuppressic acid **312**, followed by treatment with 1,1'-(Azodicarbonyl)dipiperidine (ADDP) under Mitsunobu reaction conditions to etherify the allylic alcohol, generating compound **314**. Next, compound **314** was exposed to Lewis acid Iron trichloride, which mediated the key acid catalyzed reaction that facilitated the C-O to C-C [3,3]-Claisen rearrangement, followed by ring closure to afford compound **316** as the major product as shown in Scheme 26. Benzyl deprotection with Pd/C provided STR-1 (1**84a**), and further deprotection of the methoxy group led to STR-3 (**184b**). Alternatively, **184a** could be subjected to amination conditions to yield compound **317**, which was further functionalized with lead tetraacetate (LTA) and iodine-promoted hypoiodite reaction under photolysis to provide the di-iodo compound **318**. Compound **318** was set for an intramolecular cyclization under basic conditions with elimination of the other iodo-group to provide 1,2-didehydro-STR-9 (**184c**) as the unique intermediate, which was reduced under Pd/C to provide STR-9 (**184d**). Next removal of the methoxy-protection group resulted in the natural product STR-2 (**184**) in good yields.

**Scheme 27:**
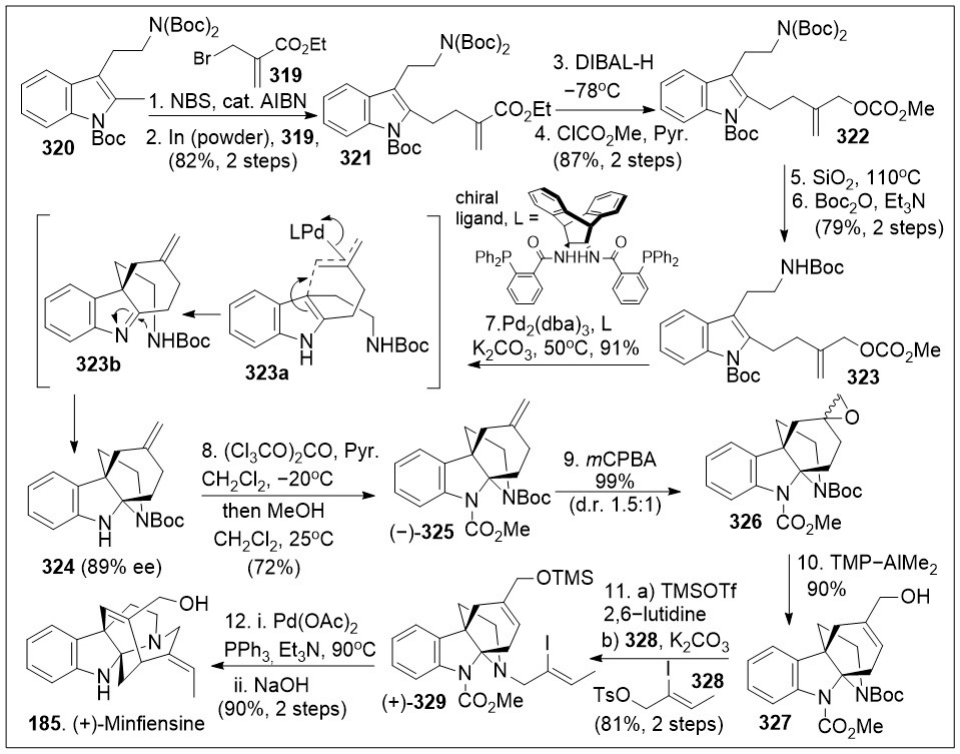
Total synthesis of monoterpene indole alkaloid (+)-minfiensine.

Finally, a selected number of monoterpene indole alkaloids, which represent a large group of more than 3000 different compounds and are mainly present in three families: *Rubiaceae*, *Apocynaceae*, and *Loganiaceae* will be included in this review. Numerous phytochemical studies have led to the discovery of potent bioactive molecules as exemplified by the anti-cancer natural product vinblastine. Minfiensine, a secoiridoid indole alkaloid-type, which was isolated from the African plant *Strychnos minfiensis* by Massiot and co-workers in 1989 with no structural precedence at the time has since attracted various synthetic chemists. Extracts containing these meroterpene alkaloids are used throughout the world in the practice of traditional medicine, with some heightened interest in the medicinal potential of the pure natural products. A recent short total synthesis of monoterpene alkaloid (+)-minfiensine (**185**) via an asymmetric palladium mediated cyclization reaction was reported [93]. The total synthesis is depicted in Scheme 27, and it commenced with the coupling reaction of readily available allylic halide **319** and methyltryptamine **320**. The 2-step transformation took place via a *N*-bromosuccinimide (NBS) mediated radical methyl-bromination of **320**, followed by subsequent coupling with the allyl indium intermediate derived from the allylic halide **319** to provide compound **321**, which upon DIBAL-H reduction and subsequent esterification produced allyl carbonate **322**. Protecting group maneuver of compound **322** provided intermediate **323**, which is subjected to an indole dearomative asymmetric allylic alkylation reaction mediated by Palladium (II) and the shown chiral ligand. The tetracyclic product **324** was generated in acceptable enantiomeric excess and good yields, and was *N*-protected, followed by epoxidation with *m*CPBA to afford a mixture of diastereomers of epoxide **326**. Allylic alcohol **327** was subsequently generated from epoxide **326** via an epoxide ring-opening mediated by 2,2,6,6-Tetramethylpiperidine-dimethyl Aluminum complex (TMP-AlMe_2_). Protection of the resultant alcohol with a silyl group followed by coupling with vinyl iodide **328** under basic condition provided *N*-alkylated intermediate **329**. Use of a Pd(OAc)_2_-catalyzed intramolecular Heck-type reaction developed by Overman and Wrobleski for this molecule, followed by a spontaneous β-elimination reaction generated a polycyclic intermediate. This intermediate was deprotected under basic conditions provided (+)-minfiensine (**185**). The synthesis is efficient at generating high complexity. 

**Scheme 28:**
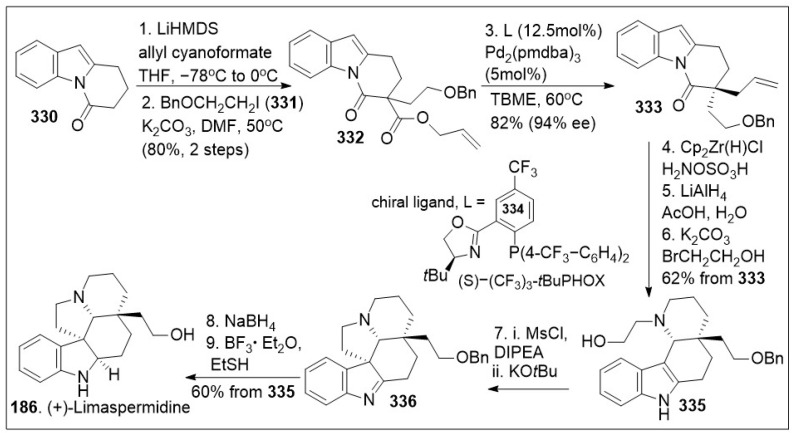
Total synthesis of (+)-limaspermidine.

The total synthesis of meroterpene indole alkaloid (+)-limaspermidine (**186**, Scheme 28) was completed in a unified approach to demonstrate the power of palladium mediated cyclization reactions [94]. Scheme 28 illustrates an efficient approach to this natural product from β-amidoester **332**, which was generated from sequential double C-alkylation of dihydropyrido[1,2-a]indolone **330** first alkylation with allyl cyanoformate in the presence of LiHMDS, and second alkylation using (2-benzyloxy)ethyl iodide **331** under basic condition reactions. Then, decarboxylative asymmetric allylation of compound **332** was mediated by Pd_2_(pmdba)_3_ with a chiral ligand to provide compound **333** featuring the required α-quaternary all-carbon stereocenter in good enantioselective excess and chemical yields. Next, compound **333** was subjected to a formal anti-Markovnikov hydroamination reaction to generate the corresponding primary amine, which was directly treated with LiAlH_4_ and quenched with aqueous acetic acid to promote intramolecular indole-iminium cyclization reaction. This furnished the *cis*-fused tetracyclic system followed by chemoselective piperidine *N*-alkylation to afford compound **335**. Compound **335** was converted to polycyclic imine **336** via 2-step pyrrolidine annulation by generating the leaving group, followed by base treatment. Subsequent NaBH_4_ mediated reduction of polycyclic imine **336** and benzyl deprotection provided the natural product (+)-limaspermidine **186** in overall good yields. 

**Scheme 29:**
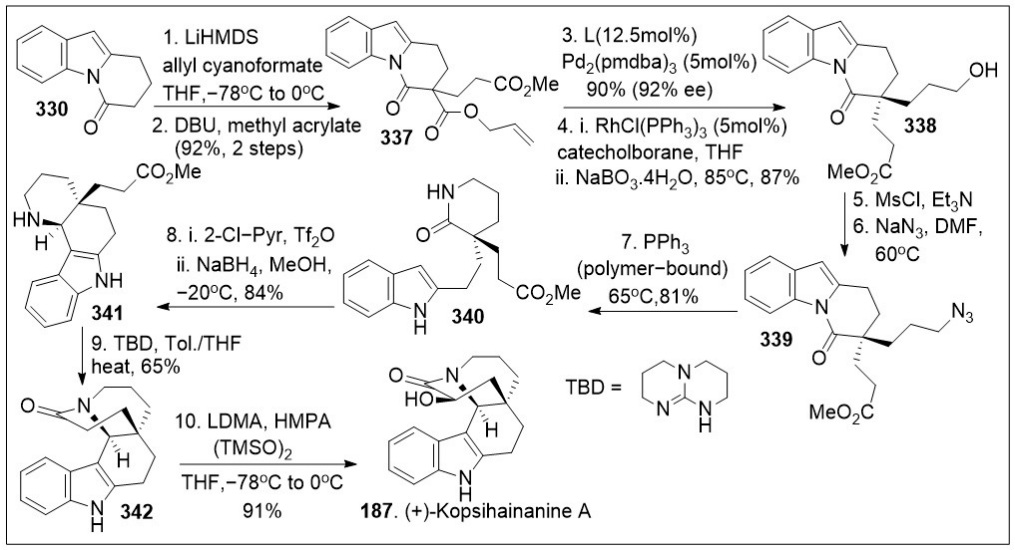
Total synthesis of (+)-kopsihainanine A.

In parallel, other similar synthetic strategies to this family of meroterpenoids with alkaloid features have been reported [94]. The total synthesis of (+)-kopsihainanine A (**187**) is shown in Scheme 29 and begins with β-amidoester **337**, which was generated via sequential double α-C-alkylation of dihydropyrido[1,2-a]indolone **330**, using allyl cyanoformate in the presence of LiHMDS. This was followed by treatment with methyl acrylate **331** under basic conditions. Further decarboxylative asymmetric allylation of compound **337** in the presence of Pd_2_(pmdba)_3_ with a chiral ligand provided an intermediate with the α-quaternary all-carbon stereocenter, which underwent Rh-catalyzed hydroboration to yield alcohol **338**. Compound **338** was mesylated and displaced by the azide group to generate compound **339**, followed by Staudinger reduction mediated by polymer-bond PPh_3_ with concomitant translactamization, which provided *δ*-lactam **340**. Subsequent Bischler-Napieralski cyclization of *δ*-lactam **340** furnished *trans*-fused tetracycle **341**. This was subjected to intramolecular lactamization between the piperidine nitrogen, and the pendant methyl ester group mediated by bicyclic guanidine base triazabicyclodecene (TBD) and generated pentacycle **342**. Upon α-hydroxylation mediated by lithium dimethylamide (LDMA) in the presence of HMPA, using bis(trimethylsilyl) peroxide as the oxidant successfully afforded the natural product (+)-kopsihainanine A (**187**). The overall synthetic maneuvers maximize the chemical yields, and a distinctive feature of this concise synthetic endeavors is the use of palladium-catalyzed reactions to form all the carbon-carbon bonds in the transformation of simple precursors to highly elaborate natural products with promising biological properties.

**Scheme 30:**
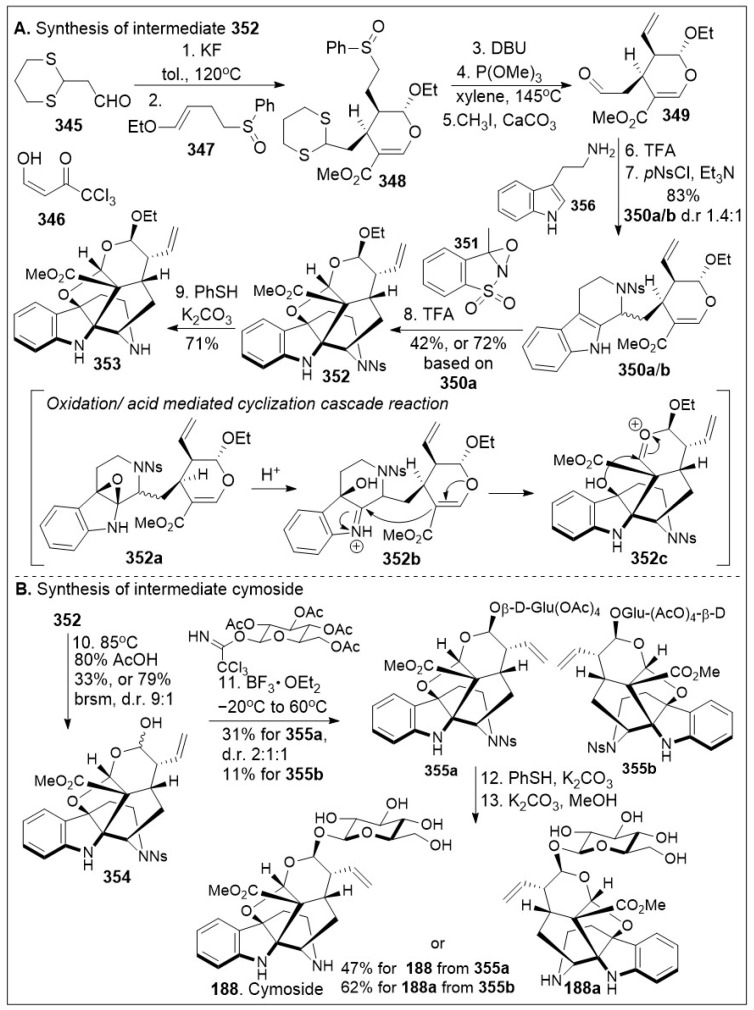
Total synthesis of cymoside via biomimetic oxidative cyclization strategy.

The total synthesis of cymoside, an oxidized derivative of strictosidine with an unprecedented hexacyclic skeleton which includes a double bridge linking the indole and the monoterpene moieties was isolated from the leaves of *Chimarrhis cymose* and provides a fertile ground for organic chemists as shown in Scheme 30. The total synthesis of cymoside (**188**) via biomimetic oxidative cyclization was reported [95]. The synthesis commenced with Knoevenagel condensation reaction between aldehyde **345** and 3-formyl-1,1,1-trichloroacetone **346** which generated enone intermediate, which upon inverse-demand hetero-Diels-Alder (HDA) reaction with enol ether **347** furnished cyclic intermediate **348** together with other diastereoisomers. Upon methanolysis and sulfoxide elimination, intermediate **348** was further converted to olefin intermediate, followed by deprotection furnished Tietze secologanin aglycon **349**. Subsequent Pictet-Spengler reaction between the secologanin aglycon **349** and tryptamine **356**, followed by protection of the secondary amine furnished the mixture of the protected strictosidine aglycon ethyl ether **350a** and its epimer **350b**. The biomimetic cascade reaction sequence of oxidation with oxaziridine **351** and acid catalyzed cyclization reaction of strictosidine aglycon ethyl ether **350a** furnished the complete framework **352** of cymoside via intermediate **352a**, **352b**, and **352c**, which was deprotected to generate ethyl ether aglycon **353** of cymoside. Hydrolysis of acetal **352** provided hemiacetal **354**, which upon Schmit glycosylation with glycosyl trichloroacetimidate, generated glycosylated product **355a** with its diastereoisomer, together with glycosylated product **355b**. Global deprotection of intermediate **355a** furnished the natural product cymoside **188**, global deprotection of compound **355b** provided product **188a** as the diastereoisomer of cymoside **188**. 

## 4. Conclusions

The described monoterpene and meroterpenoid families featured herein display an array of impressive biological activities, including antioxidant/anticancer properties. Natural resources have been an essential part of human health development for centuries and it continues to serve as an invaluable source of inspiration. The medical community has made great advances in small molecule development, but there is still a heavy reliance on natural products for their pharmaceutical applications. Biological evaluation of these natural products provide the scientific community with a larger selection of tools to understand biological and chemical systems as well as a strong starting point for the production and refinement of these chemical entities as potential therapeutic agents. Additionally, the development of synthetic methodology to access these compounds is a crucial process in the effort to conduct biological studies and large-scale production and derivatization. In order to meet the challenges of generating these complex meroterpenoids, several innovative synthetic strategies were developed including modern chemoenzymatic transformations, cationic polyene cyclization reaction, dearomatization-driven polycyclization reaction, metal mediated rearrangement cyclization and photochemical transformations. 

## Data Availability

Not applicable.
